# Stoichiometry-Induced Band Gap Opening in Epitaxial Degenerate Copper Sulfide Thin Films

**DOI:** 10.1021/acs.jpclett.6c00823

**Published:** 2026-06-07

**Authors:** Diyar Mousa Othman, Chenguang Wang, Joshua de Boer, Andrea Cicconardi, Qianyang Zhang, Zuhong Zhang, Josh Davies-Jones, Aisling Stewart, Julia A. Weinstein, Nathaniel J. Huáng, Philip R. Davies, Thomas Slater, Meng Li, Giorgio Divitini, Quan Lyu, Bo Hou

**Affiliations:** † School of Physics and Astronomy, 385187 Cardiff University, Cardiff CF24 3AA, United Kingdom; ‡ School of Chemistry, 385187 Cardiff University, Cardiff CF10 3AT, United Kingdom; § Istituto Italiano di Tecnologia, via Morego 30, Genoa 16163 Italy; ∥ Department of Physics, University of Genoa, via Dodecaneso 33, Genoa 16146, Italy; ⊥ National Physical Laboratory, Teddington TW11 0LW, United Kingdom; # Key Lab for Special Functional Materials of Ministry of Education, School of Nanoscience and Materials Engineering, Henan University, Kaifeng 475004, P. R. China; ∇ Department of Chemistry, The University of Sheffield, Sheffield S3 7HF, United Kingdom; ○ Cambridge Research Centre, Huawei Technologies Research & Development (U.K.) Ltd, Cambridge CB4 0FY, United Kingdom

## Abstract

Controlling the crystal phase and electronic structure of copper sulfide remains challenging due to its narrow stability window and sensitivity to sulfur stoichiometry, limiting reproducible optoelectronic performance. Here we report a low-temperature sulfur adsorption–corrosion strategy that converts 12–15 nm evaporated Cu into continuous copper sulfide thin films with tunable composition and electronic structure. Increasing the sulfurization time (10–60 s) reduces the Cu/S ratio from 2.4 to 1.2 and induces a phase transition from orthorhombic chalcocite-like Cu_2_S to hexagonal covellite CuS. Optical spectroscopy reveals band gap opening in sulfur-deficient films with indirect gaps of 1.15–1.41 eV. Ultraviolet photoelectron spectroscopy and Kelvin probe force microscopy show the Fermi level shifting toward the valence band, accompanied by a semiconductor-to-semimetal transition. Photoconductors based on lightly sulfurized films exhibit a wavelength-selective photoresponse and linear current transfer under 405 nm illumination, with a spectral cutoff consistent with the indirect band gap, highlighting the potential for integrated photodetectors and optocouplers.

## Graphical abstract

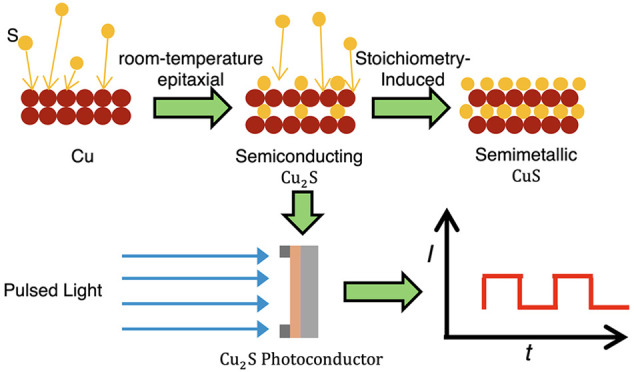

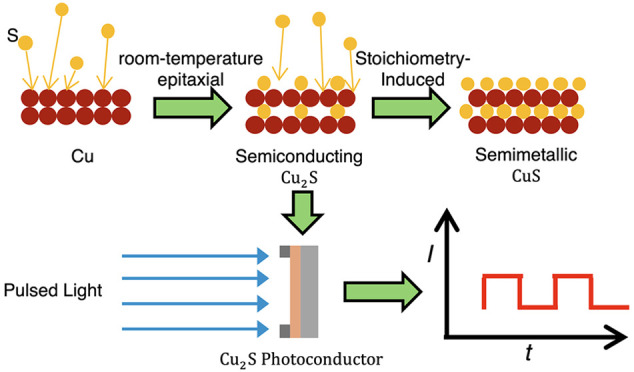

Low-dimensional transition-metal chalcogenides (TMCs) exhibit electrical, optical, and chemical properties that differ markedly from those of their bulk counterparts. As a result, TMCs have been widely explored for applications in transistors, electronics, optoelectronics, and sensing technologies.^1^ Copper sulfides are an important subclass of TMCs with a general chemical formula of Cu_*x*_S_*y*_. They have been extensively investigated as p-type semiconductors for applications including solar cells,^2^ photocatalysis,^3^ and battery electrodes.^4^ Copper sulfides exhibit a wide range of stoichiometries, spanning from copper-rich Cu_2_S to sulfur-rich CuS, with numerous intermediate phases possessing distinct compositions and physical properties. At room temperature, five stable phases are commonly reported: covellite (CuS), anilite (Cu_1.75_S), digenite (Cu_1.8_S), djurleite (Cu_1.95_S), and chalcocite (Cu_2_S).^5^

Chalcocite exists in two crystallographic forms, referred to as low- and high-chalcocite, with a phase transition occurring at 105 °C. Low-chalcocite (Figure 1a) adopts an orthorhombic crystal structure, whereas high-chalcocite exhibits a hexagonal structure. In both phases, copper is generally considered to be in the monovalent oxidation state (Cu^+^).^6^ Covellite, despite its simple stoichiometry, possesses a complex crystal structure based on a hexagonal unit cell (Figure 1b). Extensive experimental and theoretical studies have been carried out to elucidate its bonding and electronic properties. The structure consists of alternating layers of CuS_3_ trigonal units, CuS_4_ tetrahedra, and S–S bonded units. A commonly accepted ionic description of covellite is given by^7^

$${\left({\mathrm{Cu}}_{Td}\right)}^{+}{\left({\mathrm{Cu}}_{T}\right)}^{+}{\left({\mathrm{Cu}}_{Td}\right)}^{2+}{\left(S_{2}\right)}^{2-}{\left(S\right)}^{2-}$$

where Cu_*Td*_ and Cu_*T*_ denote tetrahedrally and trigonally coordinated copper, respectively. Because the tetrahedral Cu–S bonds are equivalent in length, this description can be simplified to

$$[{\left({\mathrm{Cu}}_{Td}\right)}_{2}{]}^{3+}{\left({\mathrm{Cu}}_{T}\right)}^{+}{\left(S_{2}\right)}^{2-}{\left(S\right)}^{2-}$$

A variety of synthesis routes have been developed for copper sulfides, including electrochemical deposition,^8^ electroless plating,^9^ metal–organic chemical vapor deposition (MOCVD),^10^ and hydrothermal methods.^11^ However, many of these approaches are either costly or incompatible with emerging applications that require flexible and transparent substrates, which typically cannot tolerate high processing temperatures. Recently, we reported a sulfur adsorption–corrosion method for conductive covellite CuS nanosheet films, in which thin copper layers are exposed to H_2_S vapor generated from an ammonium sulfide solution.^12^ These CuS nanosheets exhibit figures of merit within the range required for transparent conductive electrodes. A summary of solution based synthesis methods for different copper sulfide phases, along with their applications, is shown in Table 1.

**Table 1:** Overview of the State of the Art Solution Based Methods for Synthesizing Different Copper Sulfide Phases, along with Their Applications

Phase	Synthesis Method	Application
CuS thin films	Adsorption–corrosion	Transparent conductive electrodes^12^
		Raman enhancement^13^
		Electromagnetic shielding^14^
Cu_2_S nanostructures	Solution submersion	Energy storage applications^15^
Iodine doped CuS thin films	Adsorption–corrosion	Transparent conductive electrodes^16^
Phosphorus doped CuS	Solution phase synthesis	Energy storage applications^17^
Cu_2_S films	Electrosynthesis	Memory switches^18^
Cu_2_S electrodes	Solution phase synthesis	Supercapacitors^19^
CuS nanoplates	Chemical precipitation	Oxidation catalyst^20^

**Figure 1:**
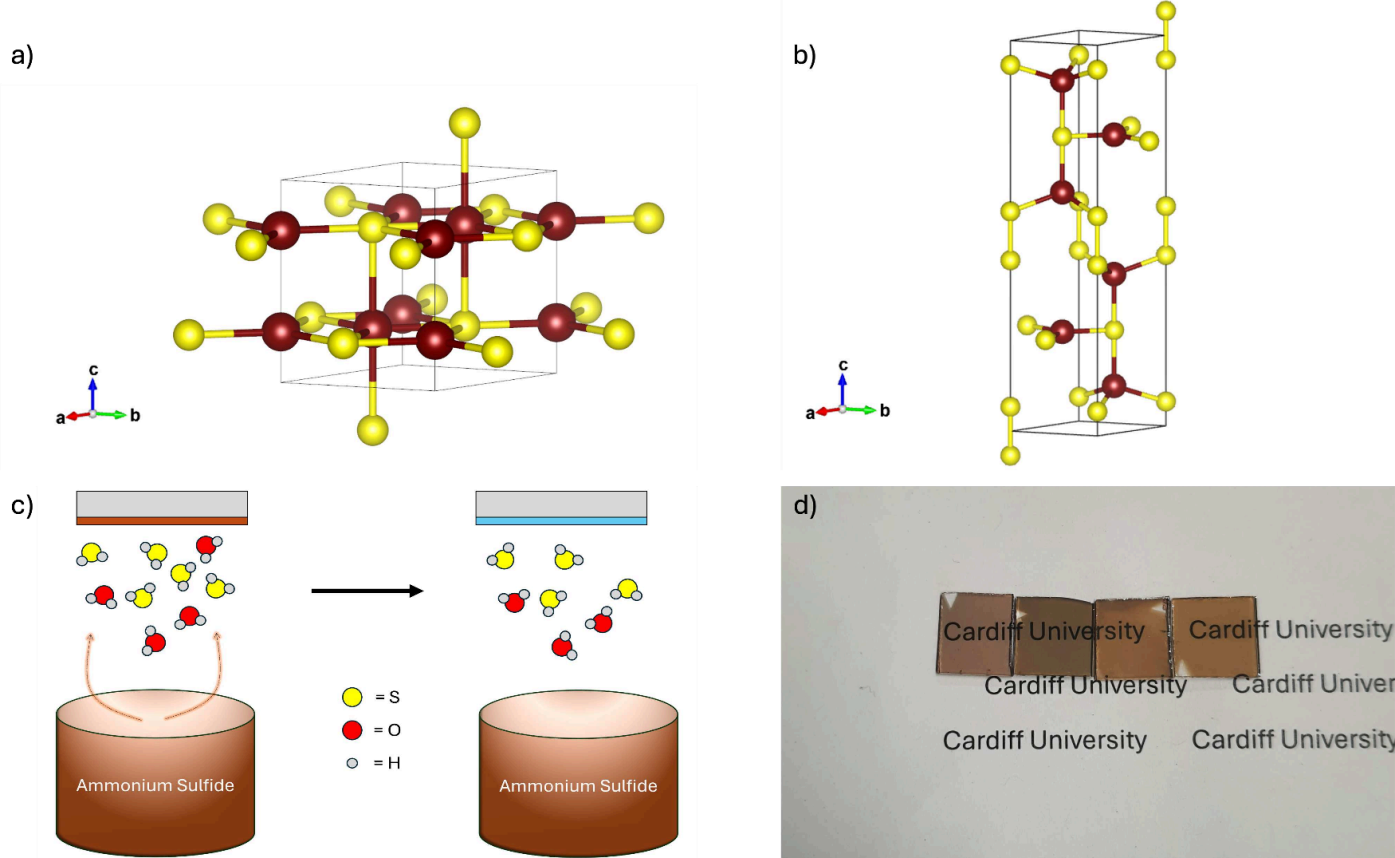
Crystal structures of (a) low-chalcocite and (b) covellite. In the ball-and-stick crystal structure models, the red spheres represent copper (Cu) atoms, while the yellow spheres represent sulfur (S) atoms. (c) The sulfurization method used in the experiments. (d) Left to right: thin films of Cu metal, 10 s sulfurized, 20 s sulfurized, and 60 s sulfurized Cu.

In the present work, we demonstrate that the adsorption–corrosion method, a room-temperature epitaxial approach, can control the formation of semiconducting chalcocite Cu_2_S and uncover direct correlations between optical transparency, composition, electrical conductivity, band-edge position, and Fermi level, enabling band gap opening in copper sulfide toward p-type transparent semiconductors.

The experimental procedure is illustrated in Figure 1c, with further details found in the Supporting Information. The metallic copper (brown) reacts with the H_2_S molecules from the ammonium sulfide solution to form copper sulfide films (blue). As the sulfurization time is increased, the sample appears bluer, especially when viewed at an angle. The color change observed as the copper (12–15 nm) films become sulfurized can be seen in Figure 1d. The reaction that takes place depends on the surface of the Cu films. Cu films oxidize when left in air, meaning the films will oxidize after being removed from the vacuum chamber after deposition. The thickness of the oxide film can reach up to 5 nm depending on the conditions.^21^

The Raman spectra of samples with different sulfurization times can be seen in Figure 2a. It can be seen that the copper oxide peaks (i.e., 220 cm^–1^)^22,23^ start disappearing as the sulfurization time increases, and by 60 s they are completely replaced by two major peaks: one at 266 cm^–1^ due to the Cu–S vibrations and the other at 474 cm^–1^ due to the S–S vibrations.^24^ The S–S vibrations are present only in sulfur-rich environments, suggesting the formation of the covellite phase or a sulfur-rich phase from the copper sulfides. The sheet resistance of the thin films for different sulfurization times can also be seen in Figure 2b. At first, there is a thin conductive film because of the copper that is present in the samples. As copper reacts with sulfur and begins to form copper sulfides, the sheet resistance increases from around >1 × 10^4^ to >1 × 10^7^ Ω/square. After a certain sulfurization time, which in this case is around 30 s, the sheet resistance plateaus at around >1 × 10^2^ Ω/square. This plateau occurs when sulfur-rich semimetallic copper sulfide phases form, as previously reported by us.^12^ The high sheet resistances occur because of the semiconducting phases of copper sulfide that form in sulfur-deficient environments. The data were fitted using a Gaussian function to show the trend in resistance; the trendline is provided as a guide to the eye and does not analytically describe the process.

**Figure 2:**
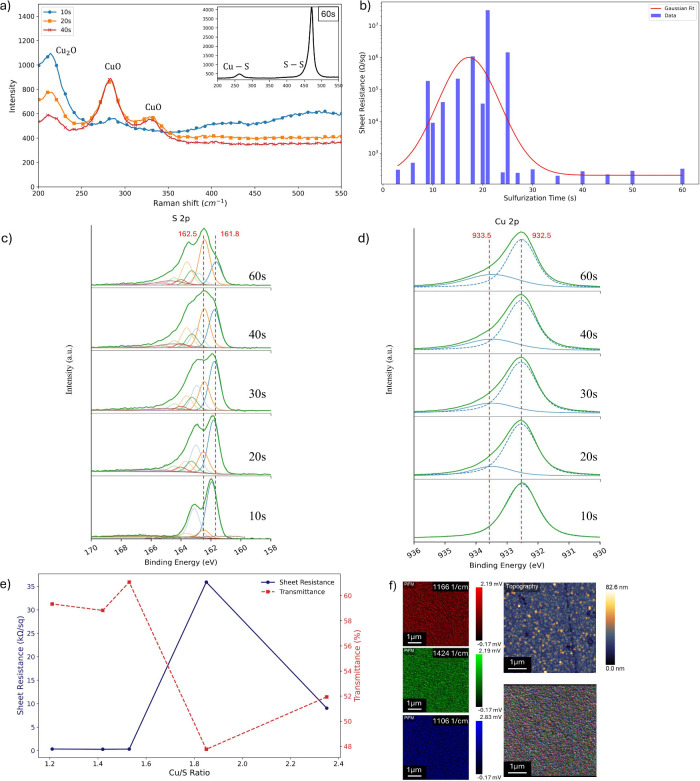
(a) The Raman spectra of samples sulfurized for different times. (b) The sheet resistance data as a function of sulfurization time fitted with a Gaussian function. (c) S 2p XPS peaks of samples sulfurized for different times. (d) Cu 2p_3/2_ XPS peaks of samples sulfurized for different times. (e) Plot of Cu/S ratio vs sheet resistance and transmittance at 555 nm for samples sulfurized for different times. (f) PiFM data showing infrared maps of the 60 s sulfurized sample at 1666 cm^–1^ (red), 1424 cm^–1^ (green), and 1106 cm^–1^ (blue), along with the AFM topography of the film (top right) and merged image (bottom right) .

To further verify the chemical states and the composition of the thin films, X-ray photoelectron spectroscopy (XPS) analysis was carried out. Figure 2c,d shows the S 2p and Cu 2p_3/2_ spectra for samples that were sulfurized for different times, respectively. The fitting was done so that the ratio of 2p_3/2_ and 2p_1/2_ peaks was 2:1, and their fwhm was the same, according to the spin–orbit splitting. The Cu 2p_3/2_ spectra have two main peaks: one around 932.5 eV and the other one around 933.5 eV. The peak at 933.5 eV appears after the 20 s sulfurization time, and the peak area increases as the sulfurization time increases. Even though the peak at 933.5 eV cannot be confidently fitted onto the 10 s sample spectra, the trend, fits, and residuals indicate that a low-intensity peak is present in the 10 s sample as well. The interpretation of these peaks is complicated, and their complexity is reflected in their descriptions in the literature. Cu 2p_3/2_ peaks from Cu_2_S and CuS have previously been reported to have the same peak position: around 932.5 eV.^25^ Other XPS databases list that Cu 2p_3/2_ peaks from CuS have been observed in the range 931.8–933.2 eV.^26^ However, it is known that 933.5 and 932.5 eV generally correspond to Cu^+2^ and Cu^+1^ states, respectively, which are both present in CuS.^27,28^ In this case, the peak intensity increase of the 933.5 eV peak and decrease in the 932.5 eV peak represent the decrease in the monovalent copper and increase in the divalent, which is an indication of the formation of covellite. There are many different contributions from different peaks in the S 2p spectra, and similarly positioned peaks have been plotted with the same color across the samples. In addition, the 2p_3/2_ and 2p_1/2_ pairs are plotted with the same color but slightly fainter, also across the different graphs, for ease. The two main peaks carrying relevant information in the S 2p spectra are around 161.8 and 162.5 eV, which originate from the sulfide and disulfide, respectively.^8,12^ The peak intensity of the sulfide decreases with the sulfurization time, whereas the disulfide peak increases. This is due to the increase in the number of S–S bonds as the transition from Cu_2_S to CuS takes place. The disulfide-to-sulfide ratio in covellite is 2:1.^29^ The peak ratio of the disulfide to sulfide increases from 0.14 for the 10 s sulfurized film to 1.96 for the 60 s sulfurized film, further suggesting the formation of the covellite structure. The peaks around 164 eV can be attributed to S_8_ or a thiol (R–SH), which should be present on the surface of the thin film. The peak at higher energies (around 167 eV) is a result of sulfate formation, also partially predicted by the molecular dynamics (MD) simulations with the adsorption of H_2_O onto the films above the H_2_S (see the MD simulations discussed in a later section). However, the counts are low, suggesting only a small amount of sulfate formation, possibly only on the surface. The Cu/S ratio for different sulfurization times can also be seen in Figure S1, where the ratio decreases from 2.4 for the 10 s sample to 1.2 for the 60 s sample, suggesting the formation of sulfur-rich copper sulfides as the sulfurization time increases.

The sheet resistance, Cu/S ratio, and transmittance at 555 nm are plotted on a 3D plot and shown in Figure 2e. The transmittance is taken at 555 nm since the human eye sensitivity is at its peak at this wavelength.^30^ As observed, the transmittance increases as the sulfurization time and S/Cu ratio increase, in addition to a decrease in sheet resistance. This transition from semiconducting to semimetallic copper sulfide phases with an increase in transmittivity makes low Cu/S ratio phases suitable for use in conductive transparent electrodes.

Photoinduced force microscopy (PiFM) has emerged as a transformative technique in nanoscale imaging, providing insights into the chemical composition and spatial organization of materials at the nanometer scale.^31^ The PiFM maps of the 60 s sulfurized sample are shown in Figure 2f. Vibrational features at 1666, 1424, and 1106 cm^–1^ are assigned to O–H,^32^ N–H,^33^ and S=O^34^ vibrational modes, respectively. The N–H and O–H signals are consistent with residual ammonium- and hydrogen-bonded species originating from the ammonium sulfide treatment.^35^ The weak S=O feature at 1106 cm^–1^ is attributed to a small fraction of oxidized sulfur species formed at the film surface, in agreement with XPS analysis and selected-area PiFM measurements with corresponding one-dimensional point IR absorption spectra (Figure S2). The homogeneous spatial distribution of these vibrational signatures indicates a uniform reaction of the Cu thin films with the ammonium sulfide.

The surface of the copper sulfide films was further studied using scanning electron microscopy (SEM). The 8 s sulfurized, 8 s sulfurized annealed, 60 s sulfurized, and 60 s sulfurized annealed thin film surfaces can be seen in Figure S3a–d, respectively. The morphology of the films after sulfurization (8 and 60 s) appears similar, with comparable grain size. The annealing was done by placing the samples on a hot plate at 180 °C. Before annealing, the grains look more disordered for both films; some cracks can be seen in the 60 s sample. While these cracks could affect sheet resistance, they do not lead to the complete breakdown of conductive paths, and the increased length of conductive paths is compensated by an increase in local conductivity. After annealing, the grains appear flatter for the 8 s sample, but a lot of pinholes can also be seen. For the 60 s sample, the film is more continuous, but some pinholes are still present. Looking closely, some layered crystals can also be observed on the 60 s annealed sample. The surface morphology of the samples was also studied with AFM. The AFM images of the 8 and 60 s sulfurized samples can be seen in Figure S4a,b, respectively. The AFM of the annealed 8 and 60 s sulfurized samples can also be seen in Figure S4c,d, respectively. The roughness of the films increases from 2.8 to 3.6 nm with the increase in sulfurization time, as well as an increase in thickness of about 3 to 4 nm. The local composition and crystal nature of the films were studied using TEM. Scanning TEM (STEM) high angle annular dark field (HAADF) images are reported in Figure 3a,b, together with elemental maps from STEM-EDX (energy-dispersive X-ray spectroscopy), which show that Cu and S are homogeneously distributed. Further characterization using HAADF-STEM-EELS (electron energy-loss spectroscopy) is reported in Figure S5, indicating a profile of the Cu L edge compatible with a +1 oxidation state.

**Figure 3:**
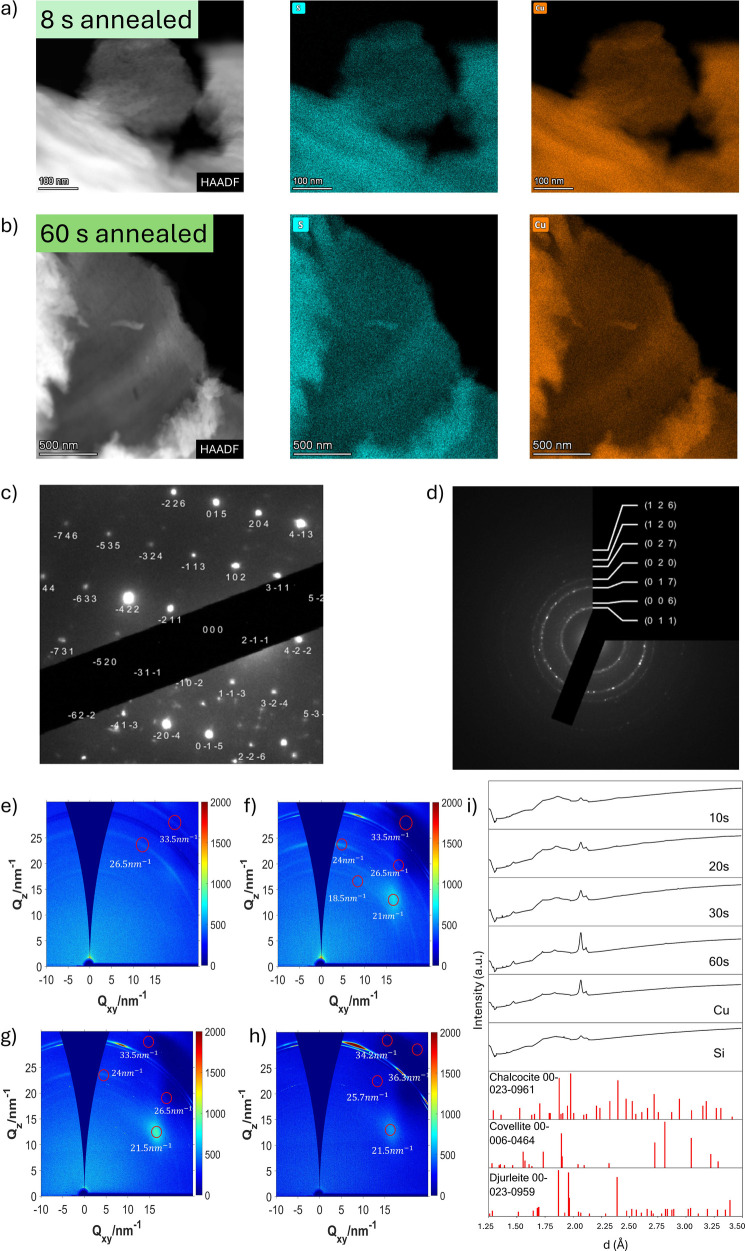
STEM-HAADF images and compositional maps from STEM-EDX for the (a) 8 s annealed and (b) 60 s annealed samples, showing the good uniformity of Cu and S. Selected-area electron diffraction patterns (SAED) for the (c) 15 s sulfurized sample and (d) 60 s sulfurized sample. The GIXRD 2D maps of the (e) 10 s sulfurized, (f) 20 s sulfurized, (g) 30 s sulfurized, and (h) 60 s sulfurized unannealed samples. (i) The GIXRD 1D peak intensities vs *d*_*hkl*_ values for samples sulfurized at different times. The intensities have been log scaled for better visualization of peaks, and the standard PDF patterns for chalcocite, covellite, and djurleite are also shown.

The SAED pattern for the samples can be seen in Figure 3c,d for the 15 and 60 s sulfurized samples, respectively. The SAED data were taken with a camera length of 300 mm, a pixel size of 0.027489 nm^–1^, a detector pixel size of 14 μm, and an acceleration voltage of 200 keV. The data were then analyzed using CrysTBox software, providing phase estimation of the copper sulfide thin films.^36^ The 15 s sample shows a strong match with the diffraction patterns of chalcocite, suggesting the presence of Cu_2_S in samples sulfurized for around 15 s. The presence of a spot pattern also suggests a relatively large grain size for this sample (see Figure S6 for comparisons with the crystallographic information file (CIF)). There are indications of the formation of covellite, although the patterns for this sample do not fit as confidently as the 15 s sample. However, it is still best matched with the covellite phase (see Figure S7 for comparisons with CIF). In addition, a ring pattern can be observed on the 60 s sample in contrast to the spot pattern for the 15 s sample, suggesting a smaller grain size and different grain orientations.

The GIXRD maps for samples sulfurized for 10, 20, 30, and 60 s can also be seen in Figure 3e–h, respectively. The approximate values of the rings/patterns observed that are originating from the Cu_2_S are labeled on the 2D maps. To analyze these data more accurately, the plots of *Q* vs intensity were used, where some peaks visible in those graphs are not very easily visible in the 2D maps, either due to the color scale or due to the *Q* value being out of range for the maps. The *Q* values can be directly converted into *d*-spacing values using the equation^37^

$$Q=\frac{2\pi }{d_{hkl}}$$ 1

The 1D GIXRD *d*-spacing vs intensity graphs are shown in Figure 3i. Several background peaks can be observed in these data; specifically, two rings that appear around 29.7 and 30.3 nm^–1^ are also very strong background peaks seen in the spectra for the Si used as the substrates for these samples. The two peaks observed in the 10 s sample have been reported for three different phases of copper sulfides previously: orthorhombic chalcocite (Cu_2_S, JCPDS no. 23-0961), monoclinic djurleite (Cu_31_S_16_, JCPDS no. 23-0959) and hexagonal Cu_2_S (JCPDS no. 26-1116), which share a lot of their main peaks with each other. Considering the TEM diffraction results, it is most likely that this sample has the orthorhombic structure of chalcocite Cu_2_S. For the 20 and 30 s samples, more peaks appear that match the data of chalcocite and djurleite, further confirming the formation of these Cu_2_S phases. For the 60 s sample, new peaks are observed that match with the hexagonal covellite (CuS, JCPDS no. 06-0464), which are not all visible on the 2D map. Three extra peaks at 38.8, 45.7, and 51.4 nm^–1^ match with the covellite data, suggesting the formation of this phase at longer sulfurization times. However, the presence of previous peaks, along with the observation of layered crystals from the SEM images, indicates that multiple phases of copper sulfides might be present in these longer sulfurized thin films.

The binding energy of the small molecules to the copper was determined by using MD simulations (see details in the Supporting Information). The binding energy results of the MD simulation can be seen in Figure 4a. The binding energies of H_2_O and NH_3_ are quite similar and on the lower end compared with those of the other molecules. O_2_ has a higher binding energy than H_2_O and NH_3_, and this result is expected since Cu oxidizes in air. However, the binding energy of H_2_S is almost two times higher than that of O_2_, which means that in an environment where both are present, the preferred reaction would be between Cu and H_2_S. An example of the simulation setup can be seen in the inset of Figure 4a, where O_2_ molecules are sent toward the surface of Cu along with H_2_S molecules. It can be seen that some O_2_ molecules remain adsorbed on the surface but mostly above the H_2_S molecules that are adsorbed before the O_2_ molecules.

**Figure 4:**
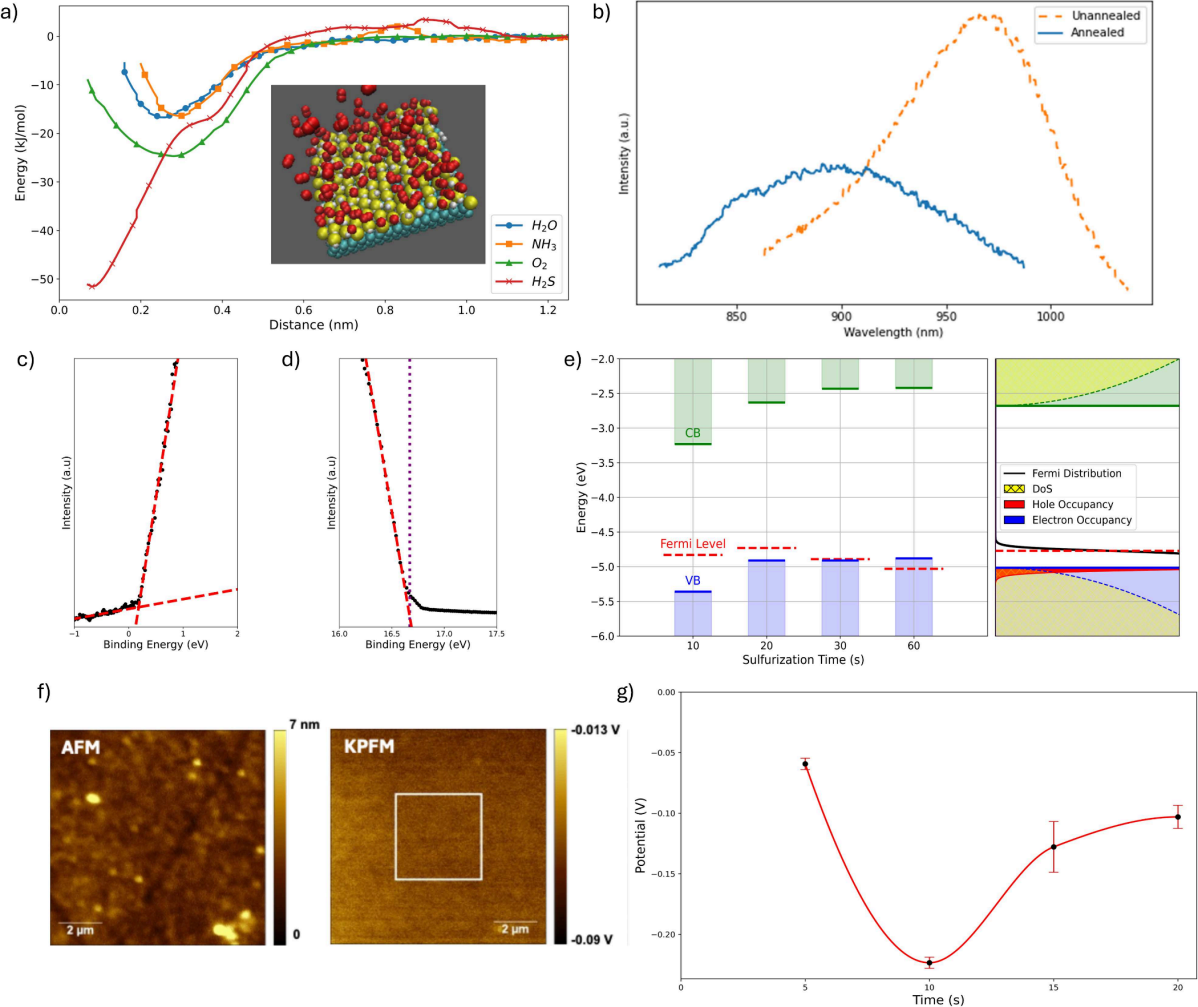
(a) The binding energy data as a function of distance from the surface, obtained from the MD simulations for different tested molecules. Inset: visual of the MD simulation for a system with 125 H_2_S and 125 O_2_ molecules (sulfur = yellow, oxygen = red, hydrogen = white). (b) The PL spectra for 8 s sulfurized annealed and unannealed samples. (c) An example of the calculation of the valence band edge using UPS data. (d) An example of the calculation of the secondary electron cutoff region. (e) The valence band maximum, Fermi level, and conduction band minimum for samples sulfurized for different times and how the hole and electron distributions look. The conduction band minimum is calculated using direct band gap values for all samples. (f) Representative AFM (left) and KPFM (right) scans of the 5 s sulfurized Cu_2_S film. (g) Sample (films sulfurized for different time scales) surface work functions with respect to the KPFM tip. The data are fitted with a piecewise cubic interpolation as a guide.

Optical properties have also been measured for the copper sulfide thin films (Figure S10). The transmittance and absorbance spectra for different sulfurization times can be seen in Figure S10a,b, respectively. The biggest change is observed between the 10 and 20 s sulfurized samples. The refractive index and extinction coefficient of the films extracted from ellipsometry measurements can also be seen in Figure S10c,d, respectively. After 20 s, a similar trend is followed with increasing sulfurization time, with the biggest change observed between the 10 and 20 s sulfurized samples. However, it should be noted that some overestimation is possible with the ellipsometry measurements due to the roughness of the thin film samples causing more scattering than expected, resulting in the detector receiving less signal. The high extinction coefficient at higher wavelengths might be a result of this or due to the intrinsic silicon substrate that was used. The direct and indirect band gaps of the thin films were also calculated using Tauc plots. An example of a Tauc plot for calculating the direct band gap of the 40 s sulfurized sample can be seen in Figure S11. The calculated band gaps can be seen in Table 2. The reported indirect band gap (*E*_g_^ind^) and direct band gap (*E*_g_^dir^) values for Cu_2_S are 1–1.5 and 1.8–2.2 eV, respectively.^38–43^ On the other hand, CuS has reported direct band gap values of 2.0–2.8 eV.^44–47^ Even though there is an indication that hexagonal structures do not have any indirect band gaps,^48^ there is no direct evidence, and hence, the indirect band gaps are generally reported for covellite as well. The band gap values calculated using the Tauc plots agree with the reported literature values. The PL data for the 8 s sulfurized and 8 s annealed thin films are shown in Figure 4b. The unannealed film has a PL peak around 970 nm, whereas the annealed film has a peak around 900 nm. It can also be seen that the PL is quenched when going from the unannealed to annealed film, as well as an increase in the fwhm of the PL spectrum. The most probable reason behind the quenching is due to the increased sulfur content and the increased conductivity.^49–51^ The PL spectrum indicates that the as-prepared Cu_2_S exhibits an indirect band structure, with a band gap of ∼1.2 eV, consistent with reported values for indirect Cu_2_S (Table 2).

**Table 2:** Indirect *E*_g_^ind^ and Direct *E*_g_^dir^ Band Gaps of Copper Sulfide Thin Films with Different Sulfurization Times, Calculated Using Tauc Plots

Sulfurization Time (s)	*E*_g_^ind^ (eV)	*E*_g_^dir^ (eV)
10	1.15	2.13
20	1.18	2.28
30	1.40	2.48
40	1.38	2.46
50	1.41	2.49
60	1.38	2.46

The valence band maximum (VBM) was determined from the intersection of baseline and rising-edge fits, and the secondary electron cutoff (SECO) was determined from the high-energy cutoff intercept (ultraviolet photoelectron spectroscopy (UPS), Figure 4c,d). As shown in Figure 4e, the energy separation between the Fermi level and the valence band progressively decreases with increasing sulfurization time. After 10 s of sulfurization, the Fermi level moves closer to the VBM but remains well within the band gap, indicating that as-prepared Cu_2_S behaves as a p-type semiconductor. As the sulfurization time increases from 20 to 60 s, the Fermi level shifts to the VBM and eventually lies below it, driving a transition from a p-type semiconductor to a semimetal. This semiconductor-to-semimetal transition is clearly resolved by our UPS measurements and density-of-states analysis, in good agreement with previous predictions for copper sulfides.^39,48^

As shown in Figure 4f,g, this trend is further supported by Kelvin probe measurements (Figures S13–S17), which determine the surface work function via the contact potential difference between the sample and KPFM tip (calibrated with a reference electrode, Al and Au on silicon, Figures S18 and S19). With increasing sulfurization time, the work function first increases and then systematically decreases relative to the reference sample, agreeing with the UPS measurements revealing a progressive downward shift of the Fermi level toward the valence band maximum (Figure S14–Figure S17).

We fabricated a two-terminal photodetector using the as-prepared Cu_2_S to evidence the opening of the copper sulfide band gap. The Cu_2_S film was deposited onto prepatterned ITO electrodes, enabling direct interrogation of photocarrier generation, transport, and recombination in a device geometry (Figure S20). Following Cu deposition (12–15 nm), the device was sulfurized for 8 s and annealed at 180 °C for 15 min. A schematic of the testing setup can be seen in Figure 5a. The device structure as well as the SEM image is shown in Figure 5b and the inset, respectively. Figure 5c presents the photocurrent response under varying incident powers of 405 nm illumination. The transient decay is well described by a quad-exponential function (Figure S22), indicating the presence of multiple carrier relaxation pathways such as trap states and nonradiative recombination channels in Cu_2_S films.

**Figure 5:**
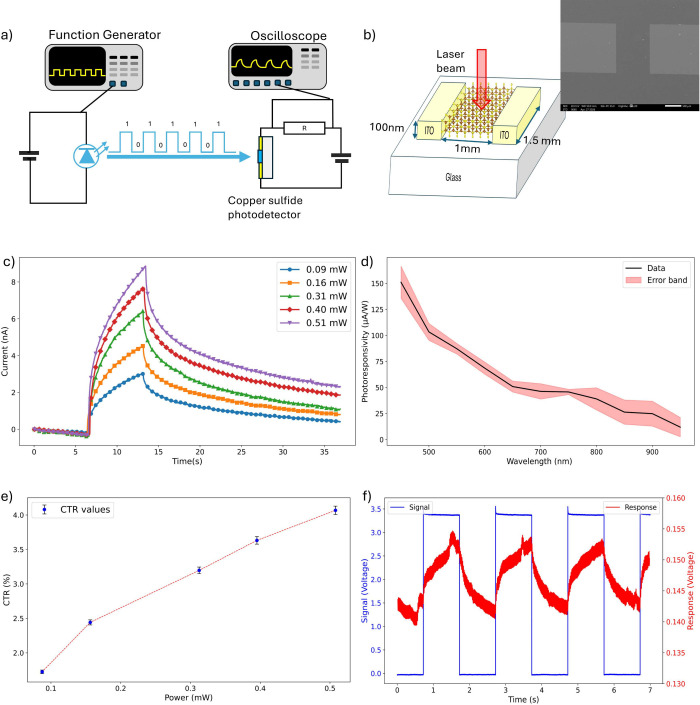
(a) Schematic showing the testing setup for the Cu_2_S (8 s sulfurized) photodetector as well as (b) the device structure and the SEM image (inset). (c) The photoresponse of the 8 s sulfurized device under 405 nm illumination through different optical filters. (d) The photoresponsivity of the device at different wavelengths. (e) The CTR values for the device for different illumination powers at 405 nm. (f) The device response to an LED blinking at 0.5 Hz.

Further evidence of semiconducting behavior is provided by the wavelength-dependent photoresponsivity shown in Figure 5d, which exhibits a pronounced spectral cutoff and approaches zero for photon energies below the band gap. This response is consistent with band-to-band optical excitation governing the photoconductive behavior and further suggests an indirect band structure nature of the as-prepared Cu_2_S with an effective band gap of approximately 1.2 eV (see Table 2). While these measurements primarily establish the device as a photodetector, the current transfer ratio (CTR) also links its performance to optocoupler functionality, representing a key optocoupler figure of merit and suggesting a potential relevance to optocoupler applications. The CTR, evaluated as a function of optical power and exposure time, is shown in Figure 5e and Figure S21, respectively. Optical power measurements were performed using 405 nm illumination, while exposure-time-dependent measurements employed 605 nm illumination. The CTR exhibits a linear dependence on the optical power across the investigated range, indicating proportional photocarrier generation and efficient charge extraction. In contrast, the CTR displays a nonlinear dependence on exposure time, reflecting the influence of carrier trapping and delayed recombination processes that dominate the transient photoresponse observed in Figure 5c. Figure 5f demonstrates the real-time response of the Cu_2_S device under modulated blue LED illumination (Figure S23), confirming reversible and reproducible semiconducting photoconductive switching.

In summary, we report a low-temperature sulfur adsorption–corrosion strategy to convert 12–15 nm evaporated Cu films into continuous copper sulfide thin films with tunable composition and electronic structure. Increasing the sulfurization time (10–60 s) decreases the Cu/S ratio from 2.4 to 1.2 and drives a phase transition from orthorhombic chalcocite-like to hexagonal covellite. Optical spectroscopy reveals band gap opening in sulfur-deficient films, with indirect and direct band gaps of 1.15–1.41 and 2.13–2.49 eV, respectively. Ultraviolet photoelectron spectroscopy (UPS) and Kelvin probe force microscopy (KPFM) show a systematic increase in work function accompanied by a semiconductor-to-semimetal transition. Photoconductors fabricated from short sulfurized films exhibit a wavelength-selective photoresponse and linear current transfer under 405 nm illumination. The wavelength-dependent photoresponse exhibits a clear spectral cutoff corresponding to the effective band gap, consistent with the extracted indirect band gap values from PL analysis and confirming the indirect band structure nature of the as-prepared Cu_2_S. One of the key challenges for Cu_2_S research is precise stoichiometry control, large area and homogeneous sulfurization. The formation of hexagonal CuS, by its saturating nature, is easier and therefore more scalable. Cu_2_S, however, needs a more controlled exposure to the hydrogen sulfide. We notice that the as-prepared Cu2S is an indirect semiconductor, and the way to change it to a direct band structure as well as the carrier concentration and transparency modulation need further explorations. It can be envisioned that Cu_2_S will be a promising material for photodetectors as well as photovoltaics applications.

## Supplementary Material



## Data Availability

Information on the data underpinning this publication, including access details, can be found in the Cardiff University Research Data Repository at 10.17035/cardiff.32475072.

## Notes

Additional experimental and computational details, including sheet resistance, XPS data, PiFM chemical mapping, SEM morphology, AFM images, STEM-EELS analysis, molecular dynamics simulations, optical characterization, band gap analysis, KPFM measurements, optocoupler testing electrical setup and device architecture, and additional optoelectronic characterization of transient response and modulation behavior (PDF)

## References

[1] Wang Y., Sarkar S., Yan H., Chhowalla M. (2024). Critical challenges
in the development of electronics based on two-dimensional transition
metal dichalcogenides. Nature Electronics.

[2] Mousavi-Kamazani M., Zarghami Z., Salavati-Niasari M. (2016). Facile and
Novel Chemical Synthesis,
Characterization, and Formation Mechanism of Copper Sulfide (Cu2S,
Cu2S/CuS, CuS) Nanostructures for Increasing the Efficiency of Solar
Cells. J. Phys. Chem. C.

[3] Tanveer M., Cao C., Ali Z., Aslam I., Idrees F., Khan W. S., But F. K., Tahir M., Mahmood N. (2014). Template free synthesis
of CuS nanosheet-based hierarchical microspheres: an efficient natural
light driven photocatalyst. CrystEngComm.

[4] Chen Y., Davoisne C., Tarascon J.-M., Guéry C. (2012). Growth of
single-crystal copper sulfide thin films via electrodeposition in
ionic liquid media for lithium ion batteries. J. Mater. Chem..

[5] Reijnen L., Meester B., de Lange F., Schoonman J., Goossens A. (2005). Comparison of CuxS Films Grown by Atomic Layer Deposition
and Chemical Vapor Deposition. Chem. Mater..

[6] Yu S., Liao R., Yang B., Fang C., Wang Z., Liu Y., Wu B., Wang J., Qiu G. (2022). Chalcocite (bio)­hydrometallurgy—current
state, mechanism, and future directions: A review. Chinese Journal of Chemical Engineering.

[7] Morales-García A., Soares A. L. J., Dos Santos E. C., de Abreu H. A., Duarte H. A. (2014). First-Principles
Calculations and Electron Density Topological Analysis of Covellite
(CuS). J. Phys. Chem. A.

[8] Li H., Wang K., Cheng S., Jiang K. (2018). Controllable Electrochemical
Synthesis of Copper Sulfides as Sodium-Ion Battery Anodes with Superior
Rate Capability and Ultralong Cycle Life. ACS
Appl. Mater. Interfaces.

[9] Chen Y.-H., Huang C.-Y., Lai F.-D., Roan M.-L., Chen K.-N., Yeh J.-T. (2009). Electroless deposition of the copper sulfide coating
on polyacrylonitrile with a chelating agent of triethanolamine and
its EMI Shielding Effectiveness. Thin Solid
Films.

[10] Nomura R., Miyawaki K., Toyosaki T., Matsuda H. (1996). Preparation of copper
sulfide thin layers by a single-source MOCVD process. Chem. Vap. Deposition.

[11] Lu Q., Gao F., Zhao D. (2002). One-Step Synthesis and Assembly of
Copper Sulfide Nanoparticles
to Nanowires, Nanotubes, and Nanovesicles by a Simple Organic Amine-Assisted
Hydrothermal Process. Nano Lett..

[12] Hong J., Kim B.-S., Hou B., Pak S., Kim T., Jang A.-R., Cho Y., Lee S., An G.-H., Jang J. E., Morris S. M., Sohn J. I., Cha S. (2021). Room Temperature
Wafer-Scale Synthesis of Highly Transparent, Conductive CuS Nanosheet
Films via a Simple Sulfur Adsorption-Corrosion Method. ACS Appl. Mater. Interfaces.

[13] Kim G. (2024). Unusual Raman Enhancement
Effect of Ultrathin Copper Sulfide. Small.

[14] Kim T., Pak S., Lim J., Hwang J. S., Park K.-H., Kim B.-S., Cha S. (2022). Electromagnetic
Interference Shielding with 2D Copper Sulfide. ACS Appl. Mater. Interfaces.

[15] Hong J., Kim B.-S., Yang S., Jang A.-R., Lee Y.-W., Pak S., Lee S., Cho Y., Kang D., Shin H. S., Hong J. P., Morris S. M., Cha S., Sohn J. I., Kim J. M. (2019). Chalcogenide solution-mediated activation protocol
for scalable and ultrafast synthesis of single-crystalline 1-D copper
sulfide for supercapacitors. Journal of Materials
Chemistry A.

[16] Pak S., Son J., Kim T., Lim J., Hong J., Lim Y., Heo C.-J., Park K.-B., Jin Y. W., Park K.-H., Cho Y., Cha S. (2023). Facile one-pot
iodine gas phase doping on 2D MoS2/CuS
FET at room temperature. Nanotechnology.

[17] Samdhyan K., Chand P., Anand H. (2023). Effective
doping of phosphorus in
copper sulfide for high performance energy storage devices. J. Alloys Compd..

[18] Sakamoto T., Sunamura H., Kawaura H., Hasegawa T., Nakayama T., Aono M. (2003). Nanometer-scale switches using copper
sulfide. Appl. Phys. Lett..

[19] Stević Z., Rajčić-Vujasinović M. (2006). Chalcocite
as a potential material
for supercapacitors. J. Power Sources.

[20] Masar M., Urbanek M., Urbanek P., Machovska Z., Maslik J., Yadav R. S., Skoda D., Machovsky M., Kuritka I. (2019). Synthesis, characterization and examination
of photocatalytic
performance of hexagonal covellite CuS nanoplates. Mater. Chem. Phys..

[21] White A. H., Germer L. H. (1942). The Rate of Oxidation
of Copper at Room Temperature. Transactions
of The Electrochemical Society.

[22] Solache-Carranco H., Juárez-Díaz G., Esparza-García A., Briseño-García M., Galván-Arellano M., Martínez-Juárez J., Romero-Paredes G., Peña-Sierra R. (2009). Photoluminescence and X-ray diffraction studies on
Cu2O. J. Lumin..

[23] Valvo M., Thyr J., Edvinsson T. (2023). Defect-Induced
Raman Scattering in
Cu2O Nanostructures and Their Photocatalytic Performance. ChemElectroChem..

[24] Isac L. A., Duta A., Kriza A., Enesca I. A., Nanu M. (2007). The growth
of CuS thin films by Spray Pyrolysis. Journal
of Physics: Conference Series.

[25] Biesinger M. C. (2017). Advanced
analysis of copper X-ray photoelectron spectra. Surf. Interface Anal..

[26] Copper (Cu), Z = 29, & Copper Compounds | The International XPS Spectra-Base of Monochromatic XPS Reference Spectra. https://xpsdatabase.com/cocopper-cu-z29/.

[27] Torres-Ochoa J. A., Cabrera-German D., Cortazar-Martinez O., Bravo-Sanchez M., Gomez-Sosa G., Herrera-Gomez A. (2023). Peak-fitting of Cu 2*p* photoemission
spectra in Cu0, Cu1+, and Cu2+ oxides: A method for
discriminating Cu0 from Cu1+. Appl. Surf. Sci..

[28] Estrada A. C., Silva F. M., Soares S. F., Coutinho J. A. P., Trindade T. (2016). An ionic liquid
route to prepare copper sulphide nanocrystals aiming at photocatalytic
applications. RSC Adv..

[29] Xie Y., Riedinger A., Prato M., Casu A., Genovese A., Guardia P., Sottini S., Sangregorio C., Miszta K., Ghosh S., Pellegrino T., Manna L. (2013). Copper Sulfide Nanocrystals with
Tunable Composition by Reduction
of Covellite Nanocrystals with Cu+ Ions. J.
Am. Chem. Soc..

[30] Wang Y., Guo H. (2021). Research Advances on Human-Eye-Sensitive Long Persistent Luminescence
Materials. Frontiers in Chemistry.

[31] Wu K.-T., El-Helou Y., Usureau E., Vuillermet E., Kazan M., Lazar M., Gautier G., Woon W.-Y., Bruyant A. (2025). Infrared photoinduced force near-field
spectroscopy
of silicon carbide. Appl. Surf. Sci..

[32] Sergeeva A. V., Zhitova E. S., Nuzhdaev A. A., Zolotarev A. A., Bocharov V. N., Ismagilova R. M. (2020). Infrared
and Raman Spectroscopy of
Ammoniovoltaite, (NH4)­2Fe2 + 5Fe3 + 3Al­(SO4)­12­(H2O)­18. Minerals.

[33] Busigny V., Cartigny P., Philippot P., Javoy M. (2004). Quantitative analysis
of ammonium in biotite using infrared spectroscopy. Am. Mineral..

[34] Al-Amin K., Kawsar M., Mamun M. T. R. B., Sahadat Hossain M. (2025). Fourier transform
infrared spectroscopic technique for analysis of inorganic materials:
a review. Nanoscale Adv..

[35] Loeffler M. J., Hudson R. L., Chanover N. J., Simon A. A. (2015). Giant-planet chemistry:
Ammonium hydrosulfide (NH4SH), its IR spectra and thermal and radiolytic
stabilities. Icarus.

[36] Klinger M., Jäger A. (2015). Crystallographic
Tool Box (CrysTBox): automated tools
for transmission electron microscopists and crystallographers. J. Appl. Crystallogr..

[37] Steele J. A. (2023). How to GIWAXS: Grazing Incidence Wide Angle
X-Ray Scattering Applied
to Metal Halide Perovskite Thin Films. Adv.
Energy Mater..

[38] Sun Z., Yi C., Hameiri Z., Bremner S. (2021). Investigation of the selectivity-mechanism
of copper (I) sulfide (Cu2S) as a dopant-free carrier selective contact
for silicon solar cells. Appl. Surf. Sci..

[39] Patel T. A., Panda E. (2019). Copper deficiency induced
varying electronic structure and optoelectronic
properties of Cu2–xS thin films. Appl.
Surf. Sci..

[40] Krylova V., Andrulevičius M. (2009). Optical, XPS and XRD studies of semiconducting copper
sulfide layers on a polyamide film /. International
journal of photoenergy..

[41] Yu Y.-X., Pan L., Son M.-K., Mayer M. T., Zhang W.-D., Hagfeldt A., Luo J., Grätzel M. (2018). Solution-Processed Cu2S Photocathodes
for Photoelectrochemical Water Splitting. ACS
Energy Letters.

[42] Saadeldin M., Soliman H. S., Ali H. A. M., Sawaby K. (2014). Optical and electrical
characterizations of nanoparticle Cu_2_S thin films. Chinese Physics B.

[43] Xu Q., Huang B., Zhao Y., Yan Y., Noufi R., Wei S.-H. (2012). Crystal and electronic structures of CuxS solar cell
absorbers. Appl. Phys. Lett..

[44] Diliegros-Godines C., Lombardero-Juarez D., Machorro-Mejía R., González R. S., Pal M. (2019). Electrical properties and spectroscopic ellipsometry studies of covellite
CuS thin films deposited from non ammoniacal chemical bath. Opt. Mater..

[45] Grozdanov I., Najdoski M. (1995). Optical and Electrical Properties
of Copper Sulfide
Films of Variable Composition. J. Solid State
Chem..

[46] Maji S. K., Mukherjee N., Dutta A. K., Srivastava D. N., Paul P., Karmakar B., Mondal A., Adhikary B. (2011). Deposition
of nanocrystalline CuS thin film from a single precursor: Structural,
optical and electrical properties. Mater. Chem.
Phys..

[47] Habiboglu C., Erken O., Gunes M., Yilmaz O., Cevlik H. C., Ulutas C., Gumus C. (2022). Effect of molar concentration on
the structural, linear and nonlinear optical properties of CuS (covellite)
thin films. Solid State Commun..

[48] Lukashev P., Lambrecht W. R. L., Kotani T., van Schilfgaarde M. (2007). Electronic
and crystal structure of Cu_2‑x_S: Full-potential
electronic structure calculations. Phys. Rev.
B.

[49] Van
Reenen S., Vitorino M. V., Meskers S. C. J., Janssen R. A. J., Kemerink M. (2014). Photoluminescence quenching in films of conjugated
polymers by electrochemical doping. Phys. Rev.
B.

[50] Divya N. K., Pradyumnan P. P. (2017). Photoluminescence
quenching and photocatalytic enhancement
of Pr-doped ZnO nanocrystals. Bulletin of Materials
Science.

[51] Babaev A. A., Sokolova A. V., Cherevkov S. A., Berwick K., Baranov A. V., Fedorov A. V., Litvin A. P. (2021). Beyond Charge Transfer: The Impact
of Auger Recombination and FRET on PL Quenching in an rGO-QDs System. Nanomaterials.

