# Supplementary material for: Stoichiometry-Induced Band Gap Opening in Epitaxial Degenerate Copper Sulfide Thin Films
Source: J Phys Chem Lett. 2026 Jun 7;17(24):6602–12. doi: 10.1021/acs.jpclett.6c00823 (PMC13288915; doi:10.1021/acs.jpclett.6c00823)
Supplement: Supplementary file 1 [file jz6c00823_si_001.pdf]

Supporting Information

Stoichiometry-Induced Band Gap Opening in Epitaxial Degenerate Copper Sulfide Thin Films

Diyar Mousa Othman[1], Chenguang Wang[1], Joshua De Boer[2], Andrea Cicconardi[3, 4], Qianyang Zhang[5], Zuhong Zhang[6], Josh Davies-Jones[2], Aisling Stewart[1], Julia A Weinstein[7], Nathaniel Huang[5], Philip R. Davies[2], Thomas Slater[2], Meng Li[6], Giorgio Divitini[3], Quan Lyu[8], and Bo Hou[1,*]

[1]School of Physics and Astronomy, Cardiff University, Cardiff, CF24 3AA, United Kingdom
[2]School of Chemistry, Cardiff University, Cardiff, CF10 3AT, United Kingdom
[3]Istituto Italiano di Tecnologia, via Morego 30, Genoa, 16163 Italy
[4]Department of Physics, University of Genoa, via Dodecaneso 33, Genoa 16146, Italy
[5]National Physical Laboratory, Teddington, TW11 0LW, United Kingdom
[6]Key Lab for Special Functional Materials of Ministry of Education, School of Nanoscience and Materials Engineering, Henan University, Kaifeng, 475004 P. R. China
[7]Department of Chemistry, The University of Sheffield, Sheffield, S3 7HF, United Kingdom
[8]Cambridge Research Centre, Huawei Technologies Research & Development (UK) Ltd, Cambridge, CB4 0FY, United Kingdom

*Email: houb6@cardiff.ac.uk

Growth of copper sulfide. Firstly, copper films were deposited with a thickness of 12-15 nm using thermal evaporation onto glass substrates, with a deposition rate of 0.2 Å/s to 0.3 Å/s. The thickness of the Cu films was controlled using a crystal monitor. The films were then held on top of an opened bottle of ammonium sulfide solution with a concentration of 40-48% in H_2O for 0 s to 60 s. A reversible reaction takes place in this environment, given in Equation 1. The H_2S evaporates from the bottle, exposing the copper films and sulfurizing them. Many different chemical reactions might take place in this interaction. Possible reactions include:[1–3]

$$(NH_4)_2S \rightleftharpoons H_2S + 2\,NH_3. \tag{1}$$

$$Cu + H_2S \longrightarrow CuS + H_2 \tag{2}$$

$$2\,Cu + H_2S \longrightarrow Cu_2S + H_2 \tag{3}$$

$$Cu_2O + H_2S \longrightarrow Cu_2S + H_2O \tag{4}$$

$$CuO + H_2S \longrightarrow CuS + H_2O \tag{5}$$

In most cases, more than one of the above reactions occurs since the thin films have different compositions across their surfaces. Furthermore, the above reactions only include Cu_2S and CuS as final products, whereas, in reality, all the intermediate phases of copper sulfides could be forming. Several different parameters affect the sulfurization process. It is important to understand these parameters to improve the experiment's quality and reproducibility. These factors include but are not limited to: (i) Thickness of Cu films; (ii) Orientation of Cu films; (iii) Concentration of H_2S gas built up inside the bottle; (iv) Distance of the samples from H_2S source; (v) Angle at which the samples are held; (vi) Surrounding pressure inside the fume hood; (vii) Temperature and humidity. For example, as the H_2S source, $(NH_4)_2S$ solution container is left closed for a

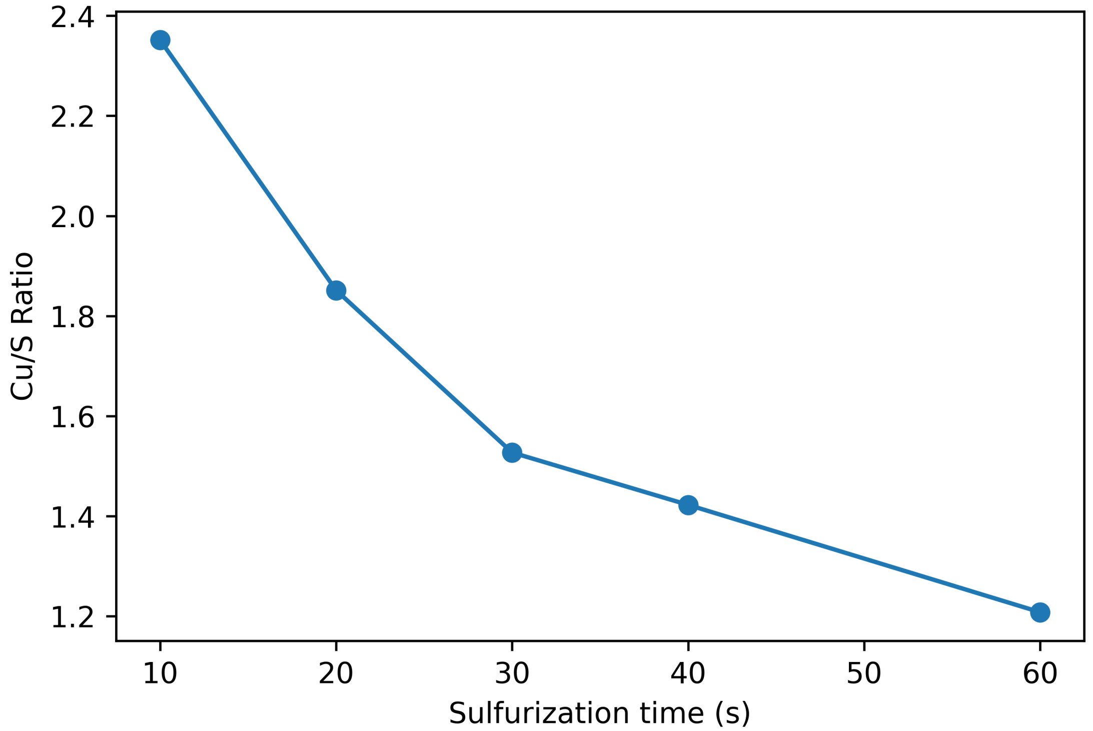

Figure S1: Cu/S atomic ratios of samples sulfurized for different durations, determined by XPS analysis.

certain amount of time, the solution and gases inside the bottle will reach an equilibrium, according to the reversible reaction given in Equation 1. As soon as the bottle is opened, the equilibrium is broken and the flux of H_2S gas starts to change with time until another equilibrium is reached. To try and account for this, the bottle was left open for 5 minutes before the sulfurization process, but some inconsistencies can still be observed.

Table S1: The fitting parameters obtained from fitting the log of sheet resistance values with a Gaussian function.

Constant	Value
A	4950 Ω/square
b	17.2 s
c	6.3 s
D	204 Ω/square

The logged data were fitted with a Gaussian distribution as

$$f(t) = A \times e^{-\frac{(x-b)^2}{(2c)^2}} + D \tag{6}$$

where A is the amplitude, D is the constant which sets the base/background, b is the mean and c is the standard deviation of the data. Because the fit was performed on logarithmically transformed data, the fitted parameters A and D were converted back to the original scale by taking 10 raised to the corresponding constants. The resulting fit parameters are summarized in Table S1. For this data set, the sheet resistance reaches a maximum at approximately 17 s of sulfurization and approaches a baseline value of around 200 Ω/square. The anomalies observed in the data may arise from several factors discussed above, most notably variations in H_2S flux, which can accelerate or retard the reaction kinetics, or differences in Cu film thickness, which alter the amount of H_2S required to form distinct copper sulfide phases.

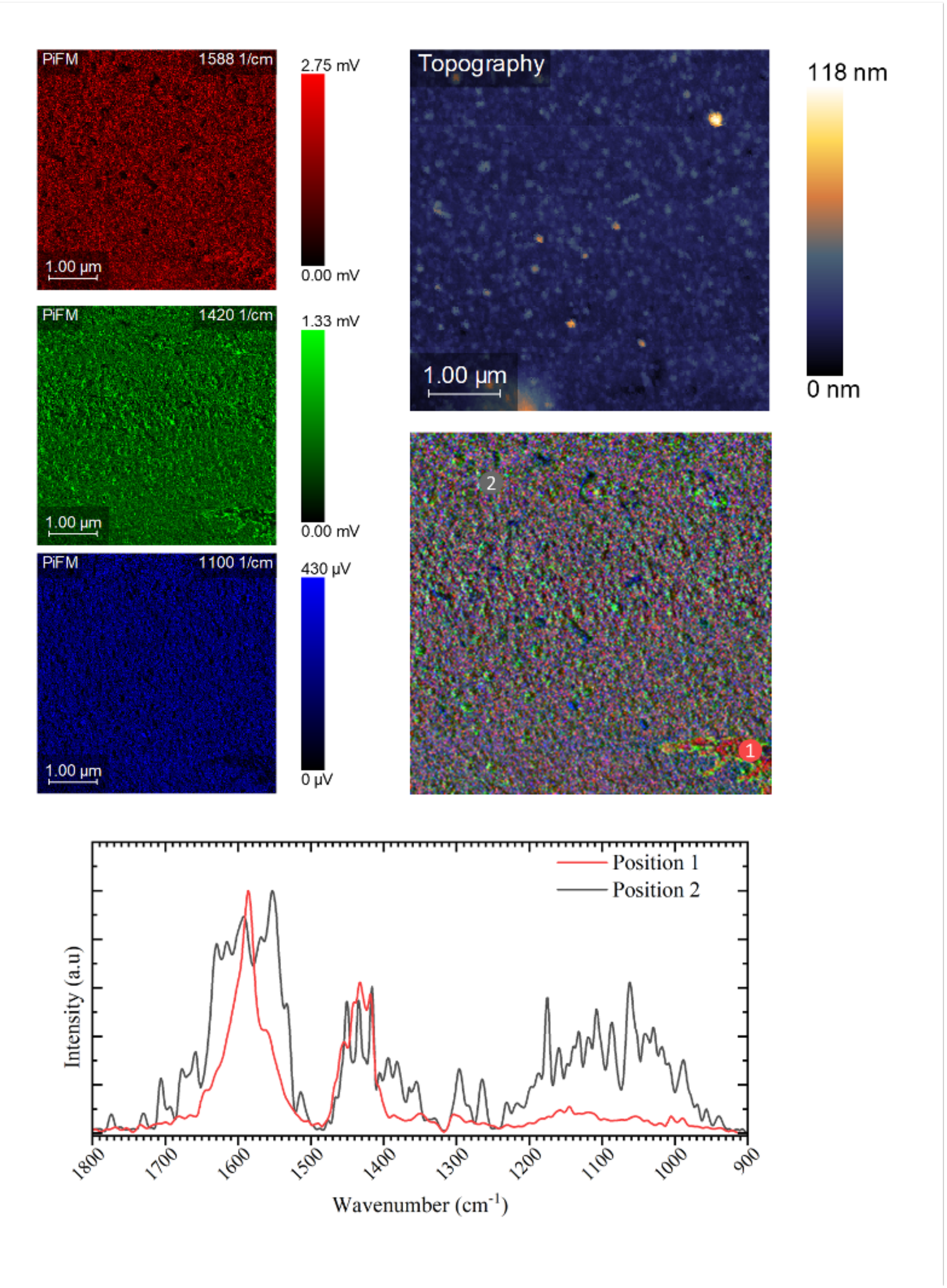

Figure S2: PiFM data of the 60 s sulfurized sample acquired at 1588, 1420, and 1100 cm^{-1}, together with the corresponding AFM topography, composite (overlay) PiFM map, and representative 1D PiFM–IR spectra. Selected area 1 shows stronger signals at 1588 and 1420 cm^{-1} with a weaker contribution at 1100 cm^{-1}, whereas selected area 2 exhibits a more homogeneous distribution of all three vibrational features. The contrast observed in selected area 1 likely reflects a localized, non-representative surface feature. The composite map is not normalized; therefore, variations in relative intensity reflect both chemical contrast and surface topography.

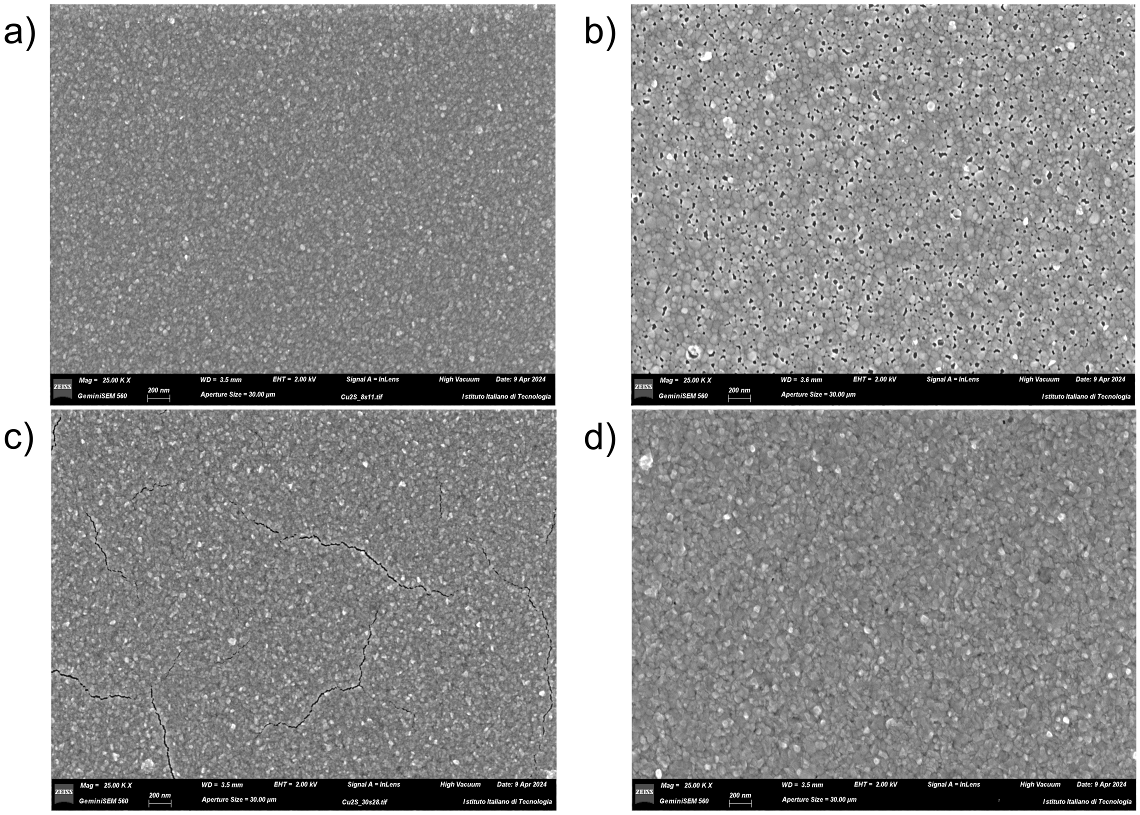

Figure S3: SEM images of (a) an 8 s sulfurized Cu_2S film, (b) an 8 s sulfurized film annealed at 180 °C, (c) a 60 s sulfurized film, and (d) a 60 s sulfurized film annealed at 180 °C.

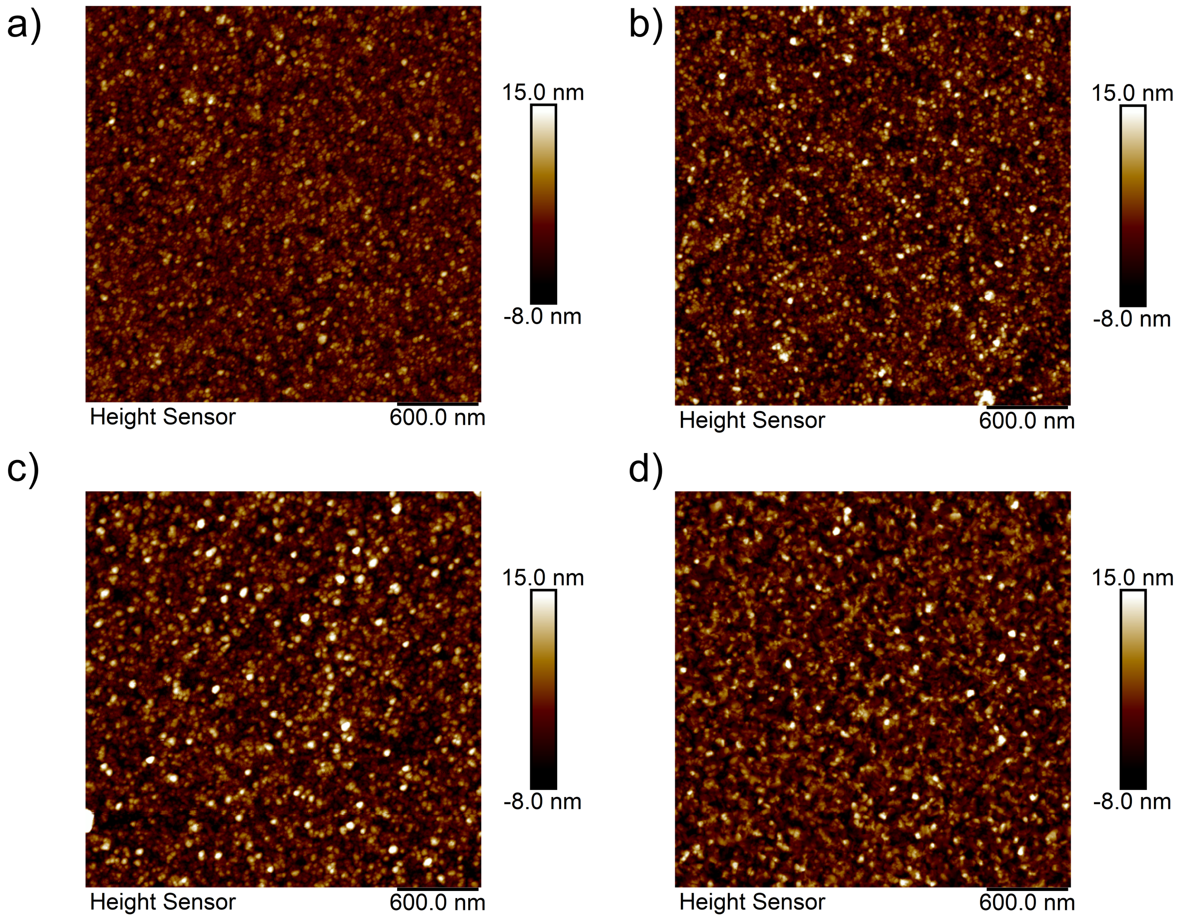

Figure S4: AFM images of (a) the 8 s, (b) the 60 s sulfurized, c) the 8 s annealed and d) 60 s annealed copper sulfide films.

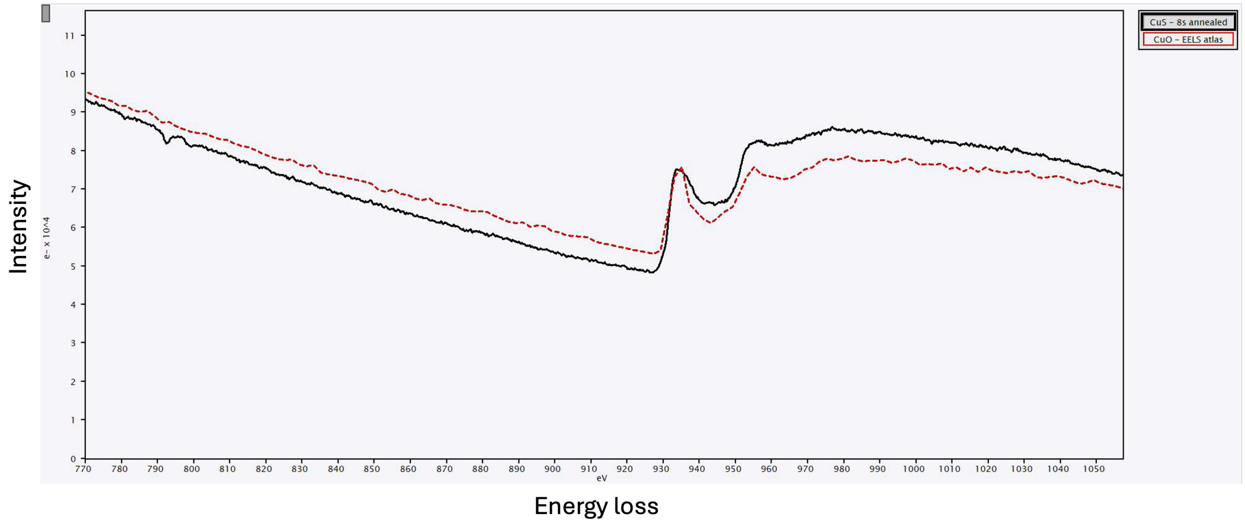

Figure S5: STEM–EELS profile of the Cu L edge for the 8 s annealed sample. The experimental spectrum (black, solid line) matches the reference spectrum (red, dashed line) of Cu_2O, consistent with a +1 oxidation state for Cu. A rigid energy shift of approximately 5 eV was applied to compensate for aberrations in the energy filter.

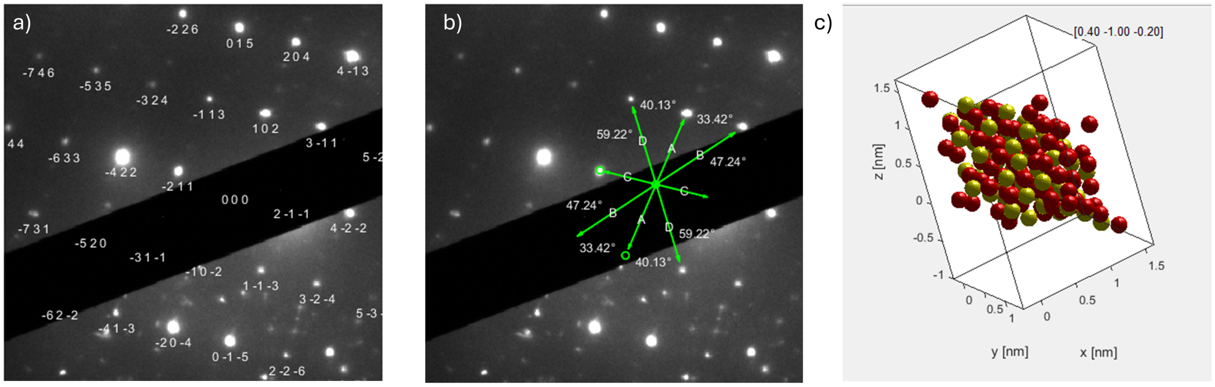

d)

Zone axis:	-2 5 1
Estimation rating:	Excellent
Consistency check:	OK
Lattice check:	OK
Total angular distance:	0.50
D-spacing STDEV:	0.0011

Vector identification	
A	1 0 2
B	-1 -1 3
C	-2 -1 1
D	-3 -1 -1

d-spacing		
	[nm]	[1/nm]
A	0.47444	2.1078
B	0.41341	2.4189
C	0.63306	1.5796
D	0.35301	2.8328

Figure S6: The measured SAED data shown with a) vector indexing and b) vector angles. c) The simulated orientation of the crystal structure. d) Tables showing the results of the comparison between the measured SAED data and Cu_2S from the crystallographic information file (CIF), the vector identification and the d-spacing values corresponding to them.

The measured SAED data shown in Figure S6 and S7 were compared to a comprehensive list of copper sulfides and the best matches were selected.

a)

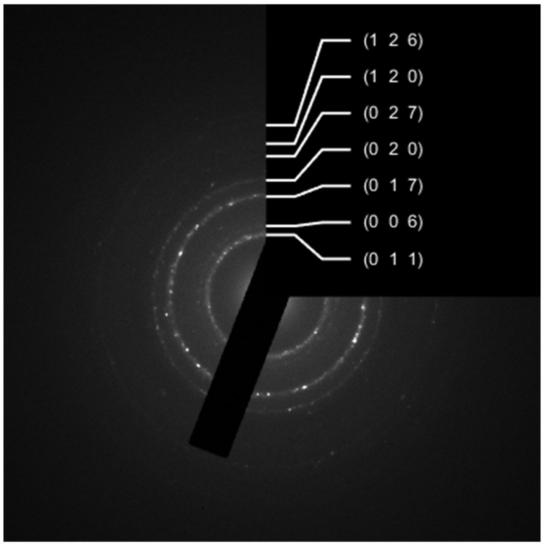

b)

Ring identification				
Plane	Radius [1/nm]		d-spacing [nm]	
	theor.	measured	theor.	measured
(0 1 1)	3.103	3.161	0.322	0.316
(0 0 6)	3.667	3.629	0.273	0.276
(0 1 7)	5.250	5.168	0.190	0.193
(0 2 0)	6.084	6.020	0.164	0.166
(0 2 7)	7.438	7.285	0.134	0.137
(1 2 0)	8.048	7.944	0.124	0.126
(1 2 6)	8.844	8.907	0.113	0.112

Figure S7: a)The measured SAED data shown with vector indexing. b) The comparison between the measured d-spacing values and the theoritical covellite obtained from CIF.

The binding energy of the small molecules to the copper was determined by using the pull algorithm of GROMACS to pull the molecule set distances from the surface, then running simulations at each of these distances and calculating the energy of the system at each separation. The reason for simulating H_2S, O_2, NH_3 and H_2O molecules is that a significant amount of these molecules is present in the reaction system. MD calculations were carried out using the GROMACS software, version 2021.5 [4,5] and run on the Welsh Supercomputer. Copper atoms were modelled using a Lennard-Jones-based potential, parameterised by Heinz et al. [6] and integrated into the CHARMM36 force field. [7] Single-molecule coordinates, partial charges and bonding parameters for H_2S, O_2 and NH_3 were obtained from the Automated Topology Builder [8] and the CHARMM force field, water was modelled using the SPC/E model. [9] The coordinates for the (111) copper surfaces were generated using the Avogadro package. [10] The copper was positioned in a simulation box of dimensions 4x4x50 nm. The metal was a layer 3 atoms thick at the centre of the z direction and filled the box in the x and y directions, with periodic boundary conditions applied in 3D space.

The simulations were carried out as follows: The copper sample underwent an energy minimisation process using the steepest descent algorithm before a constant volume (NVT) equilibration step was carried out for 100 ps at 298 K, with a 0.5 fs time step and a velocity Verlet algorithm. [11] The molecule of interest was then inserted randomly into the periodic box just above the surface, and another energy minimisation and NVT equilibration process was carried out as above, by the end of the latter of which the molecule had settled on the surface. The coordinates at this point were saved and used as the starting point for the umbrella sampling simulations to calculate the binding energy of the molecule to the copper surface.

Final atomic configurations for simulation boxes containing varying numbers of H_2S, H_2O, and O_2 molecules following 100 ps of NVT equilibration. In all cases, H_2S exhibits strong adsorption on the copper surface. O_2 bonds directly to the metal only at sub-monolayer H_2S coverages, while at higher H_2S coverages it forms an overlayer atop the completed H_2S monolayer. In contrast, H_2O shows only weak surface affinity, preferentially forming hydrogen-bonded clusters with itself rather than adsorbing on copper.

The binding energy of the small molecules to the copper was determined by using the pull algorithm of GROMACS to pull the molecule set distances from the surface, then running simulations at each of these distances and calculating the energy of the system at each separation. The first step of the pulling process was to use the "gmx_trjorder" command to find out which copper atom at the surface was initially closest to the molecule of interest (Figure S5). A harmonic pulling potential with a spring constant of $1000\,\mathrm{kJmol^{-1}nm^{-2}}$ was employed for 10 ps to pull the molecule to a set distance from the atom of interest, with pulling carried out exclusively in the z-direction, along the surface normal (Figure S8). An energy minimisation and 100 ps NVT equilibration were then carried out at 300 K and restraining the surface-molecule distance at the chosen distance. This pulling and equilibration procedure was carried out at 0.2 nm intervals for distances between 0 and 1.4 nm from the surface, and the "gmx_wham" function was used to extract a plot of the average energy of the system against surface-molecule separation for each species. Further simulations were carried out by adding different numbers of H_2S, O_2 and H_2O molecules to the box containing the equilibrated copper slab and carrying out a 100 ps NVT equilibration step as described above. Examples of the resulting systems are shown in the Figure S5 and Figure S8.

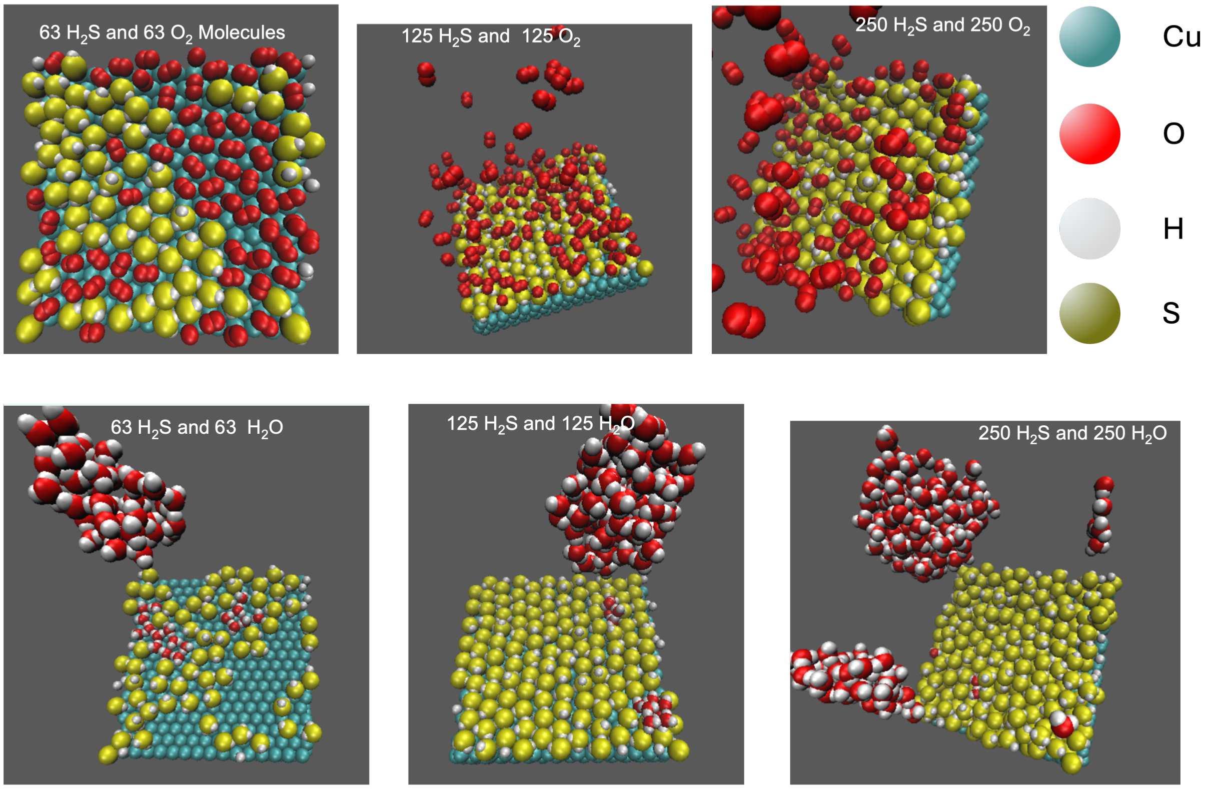

Figure S8: MD atomistic configurations illustrating the first step of the pulling process. The `gmx_trjorder` command was used to identify surface Cu atoms on the (111) facet that were initially closest to the molecule of interest.

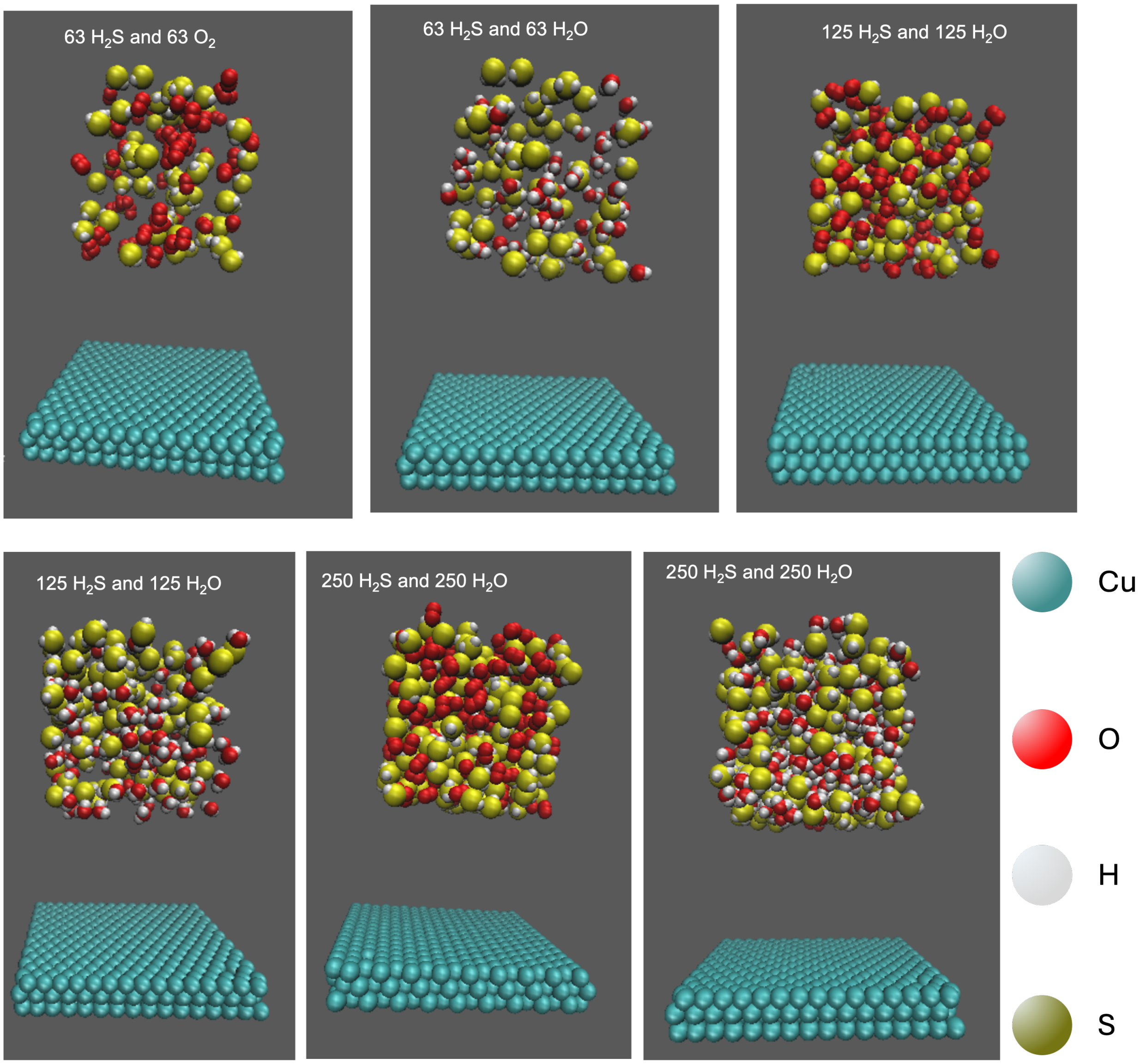

Figure S9: MD atomistic configurations illustrating the application of a harmonic pulling potential with a spring constant of $1000\,\mathrm{kJ\,mol^{-1}\,nm^{-2}}$. The potential was applied for 10 ps to pull the molecule to a defined distance from the atom of interest, with pulling carried out exclusively along the surface normal (z-direction of Cu atoms on the (111) facet).

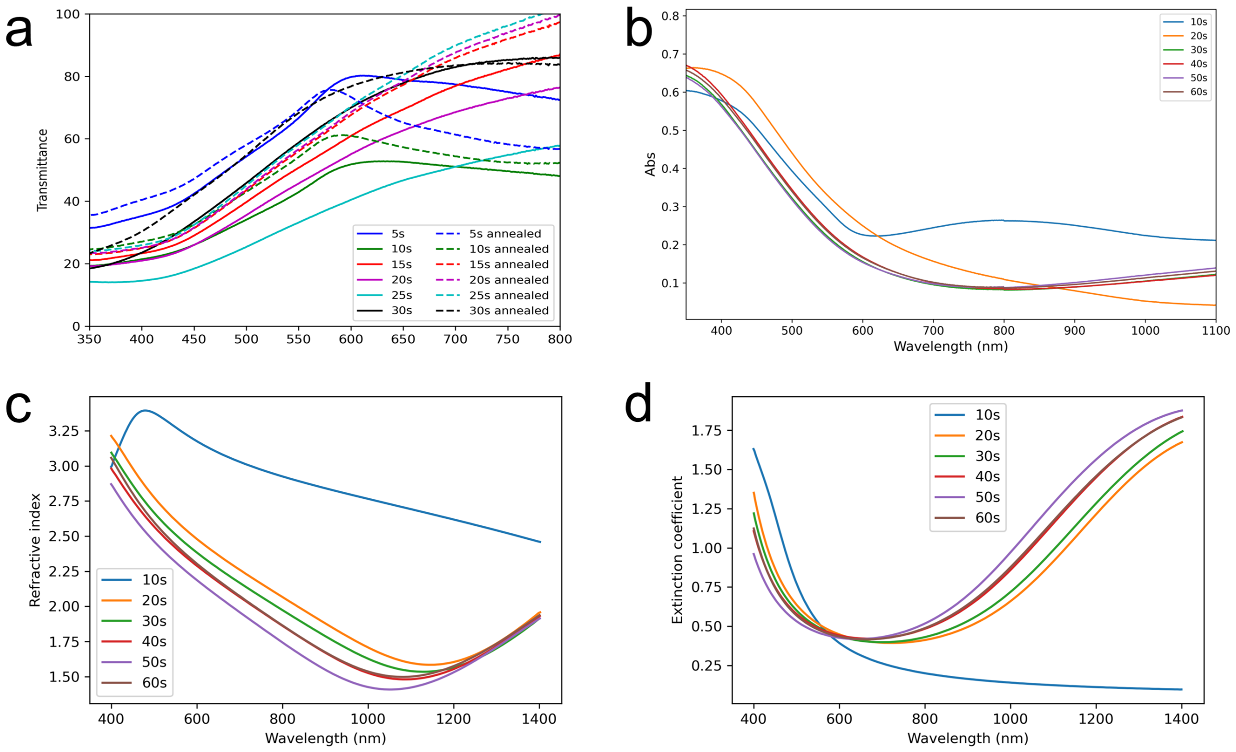

Figure S10: Transmittance (a) and absorbance (b) spectra for samples with different sulfurisation times. Refractive index (c) and Extinction coefficient (d) of samples with different sulfurization times obtained from ellipsometry measurements.

The absorption coefficient was calculated using the formula

$$\alpha = \frac{2.303}{t} A \tag{7}$$

where α is the absorption coefficient, t is the thickness of the sample and A is the absorbance at different wavelengths. This process was done for different sulfurization time samples, where $(\alpha h\nu)^2$ was used for direct band gap calculations, and $(\alpha h\nu)^{0.5}$ was used for indirect band gap calculations.

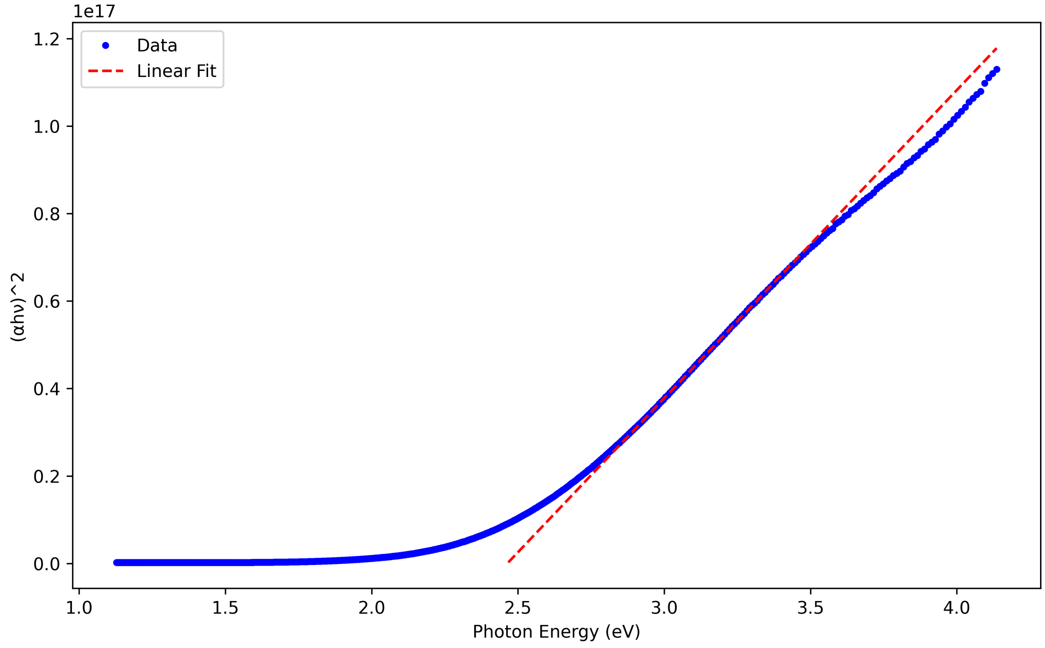

Figure S11: An example Tauc plot from calculating the direct band gap of samples, in this case the 40s sulfurized sample.

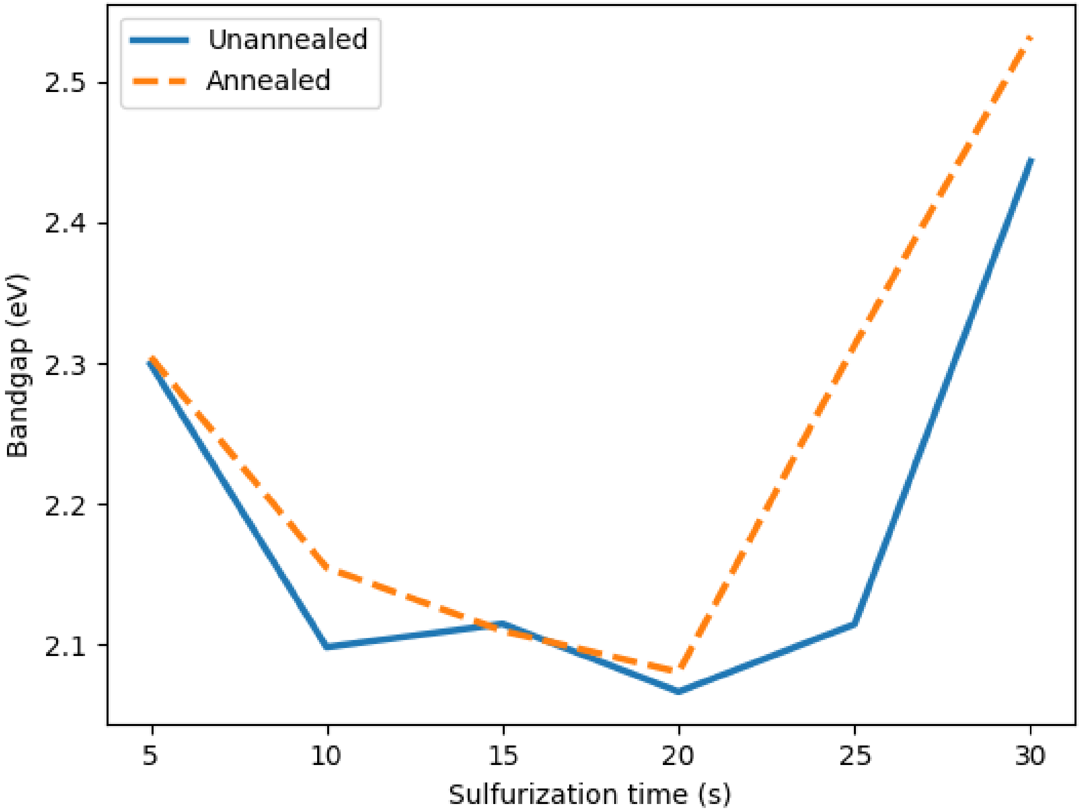

Figure S12: Optical band gaps of as-prepared Cu_2S (annealed and unannealed) as a function of sulfurzation time.

Ultraviolet Photoelectron Spectroscopy (UPS) was used to measure the valence band maximum (VBM) and the work function of the copper sulfide films. The VBM was calculated by fitting a straight line to the base of the data, fitting another straight line to the first sharp increase in intensity, and calculating the intersection of these two straight lines. This gives the VBM with respect to the Fermi level. The secondary electron cut-off (SECO) was calculated by fitting a straight line to the part of the spectrum where a sharp decrease is observed at the high energies, and calculating the x-intercept. The work function can then be calculated by using

$$\Phi = h\nu - w \tag{8}$$

where $h\nu$ is the incident photon energy (in this case He I, 21.22 eV) and w is the width of the UPS spectrum, calculated as $w = SECO - VBM$.[12] After doing the calculations, the Fermi level was calculated by subtracting the work function, and the VBM was calculated by subtracting the intersection of the two fitted lines from the Fermi level. The conduction band minimum (CBM) was then calculated by adding the direct band gap values (Figure S11) to the VBM.

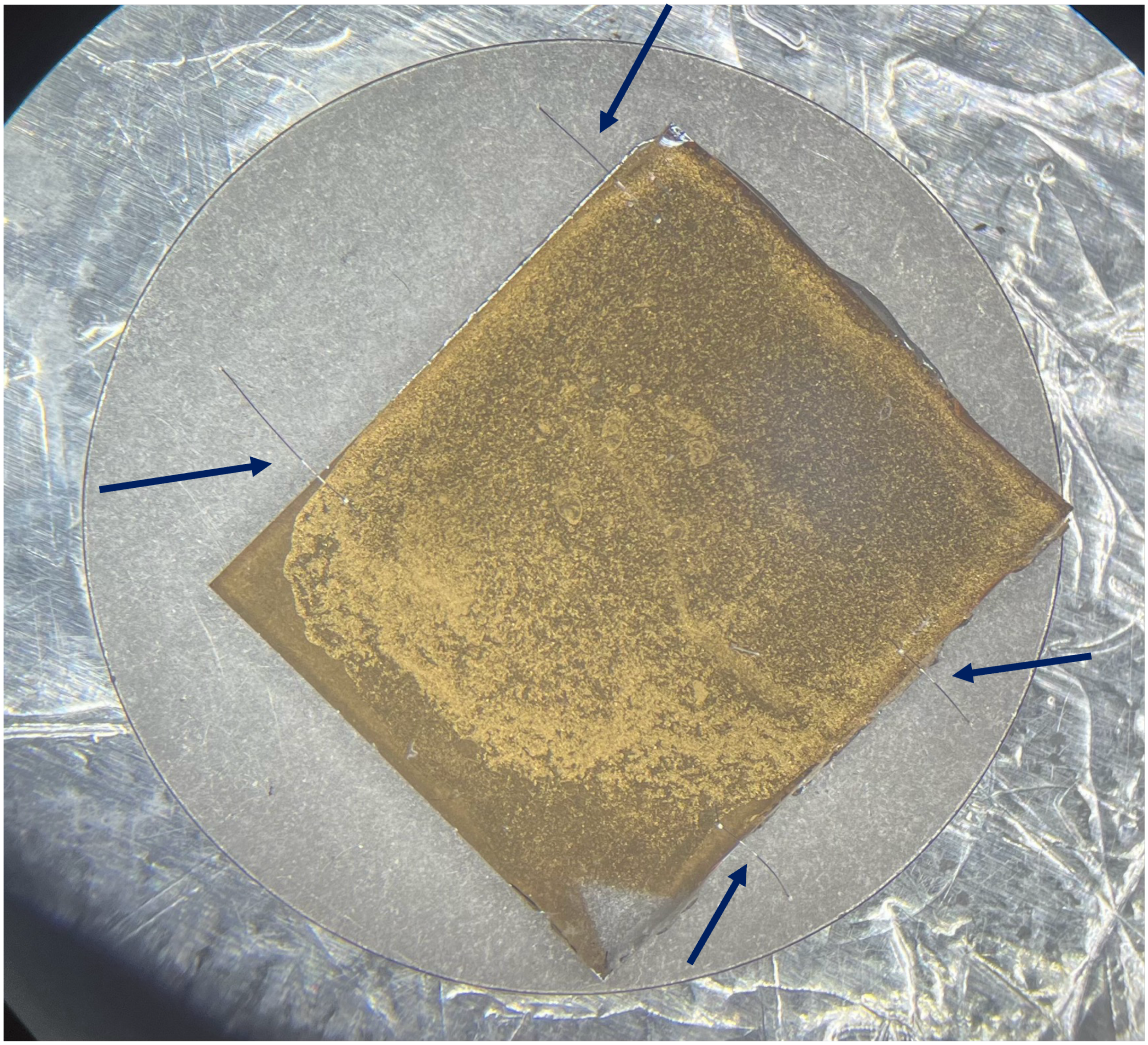

Figure S13: To perform KPFM, the sample was attached to a steel disk (red arrow) via silver paste, and the Cu_2S film was connected to the steel disk via 4 wires (blue arrows).

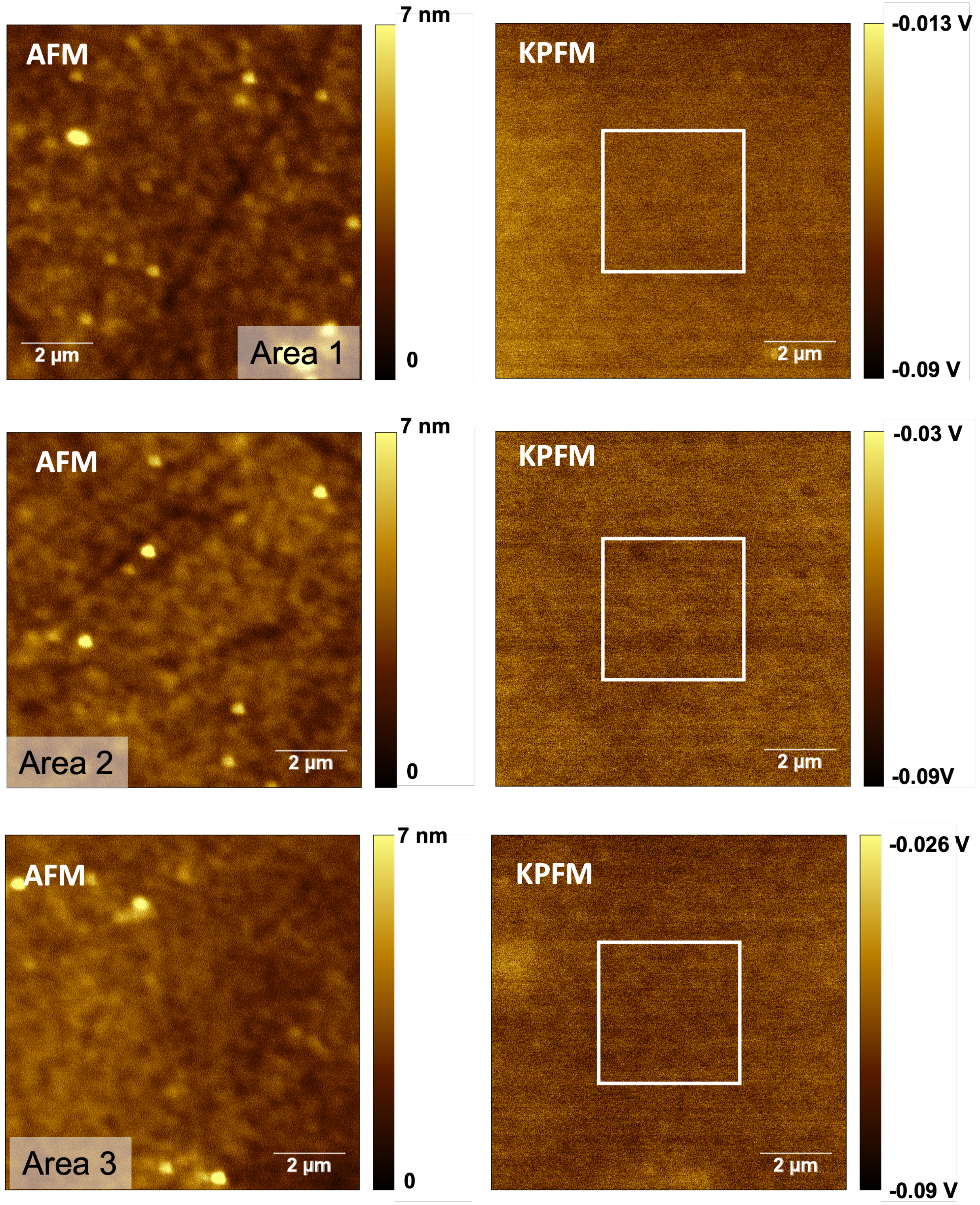

Figure S14: AFM (left) and KPFM (right) scan images of three selected areas on a 5s sulfurized Cu_2S film.

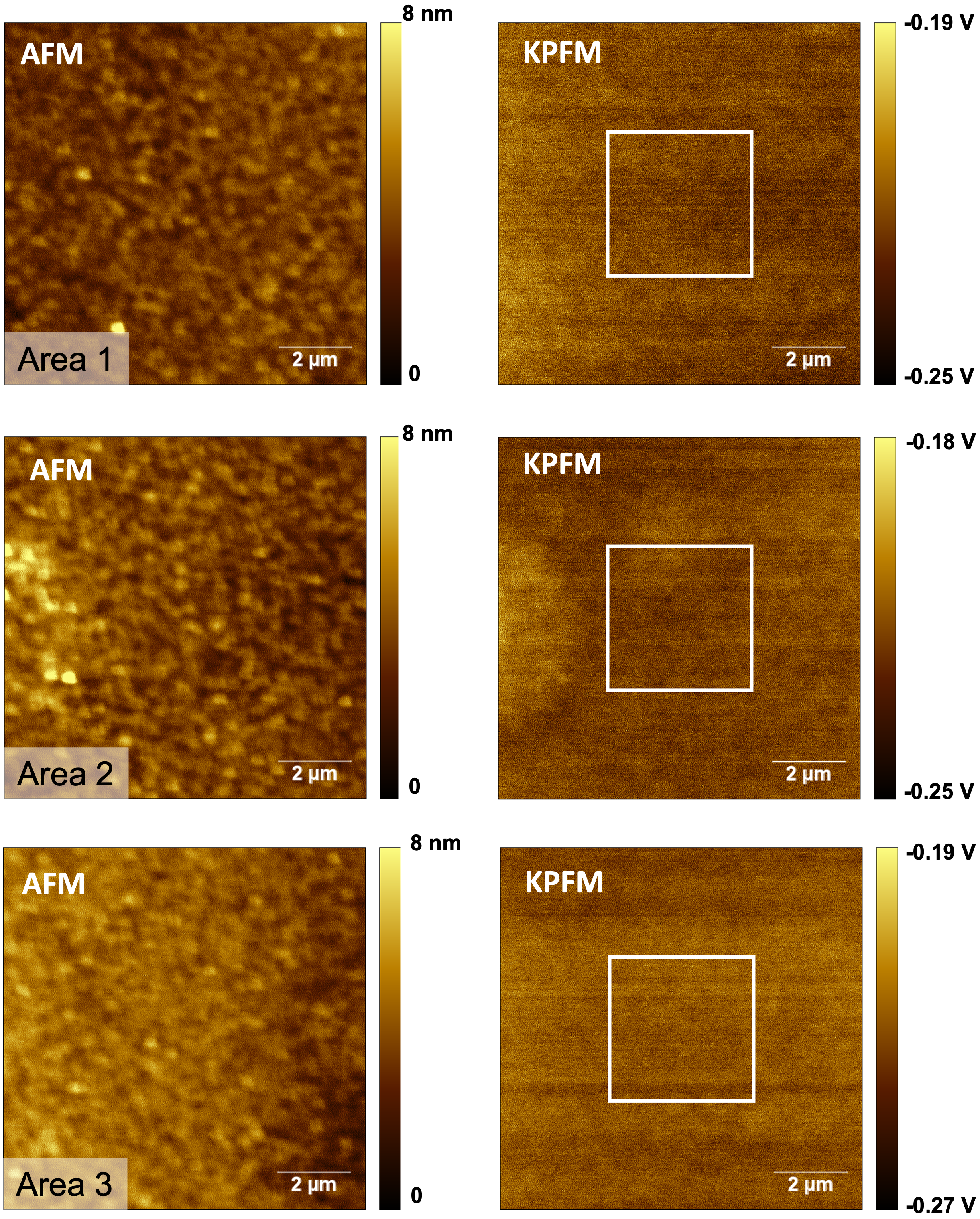

Figure S15: AFM (left) and KPFM (right) scan images of three selected areas on a 10s sulfurized Cu_2S film.

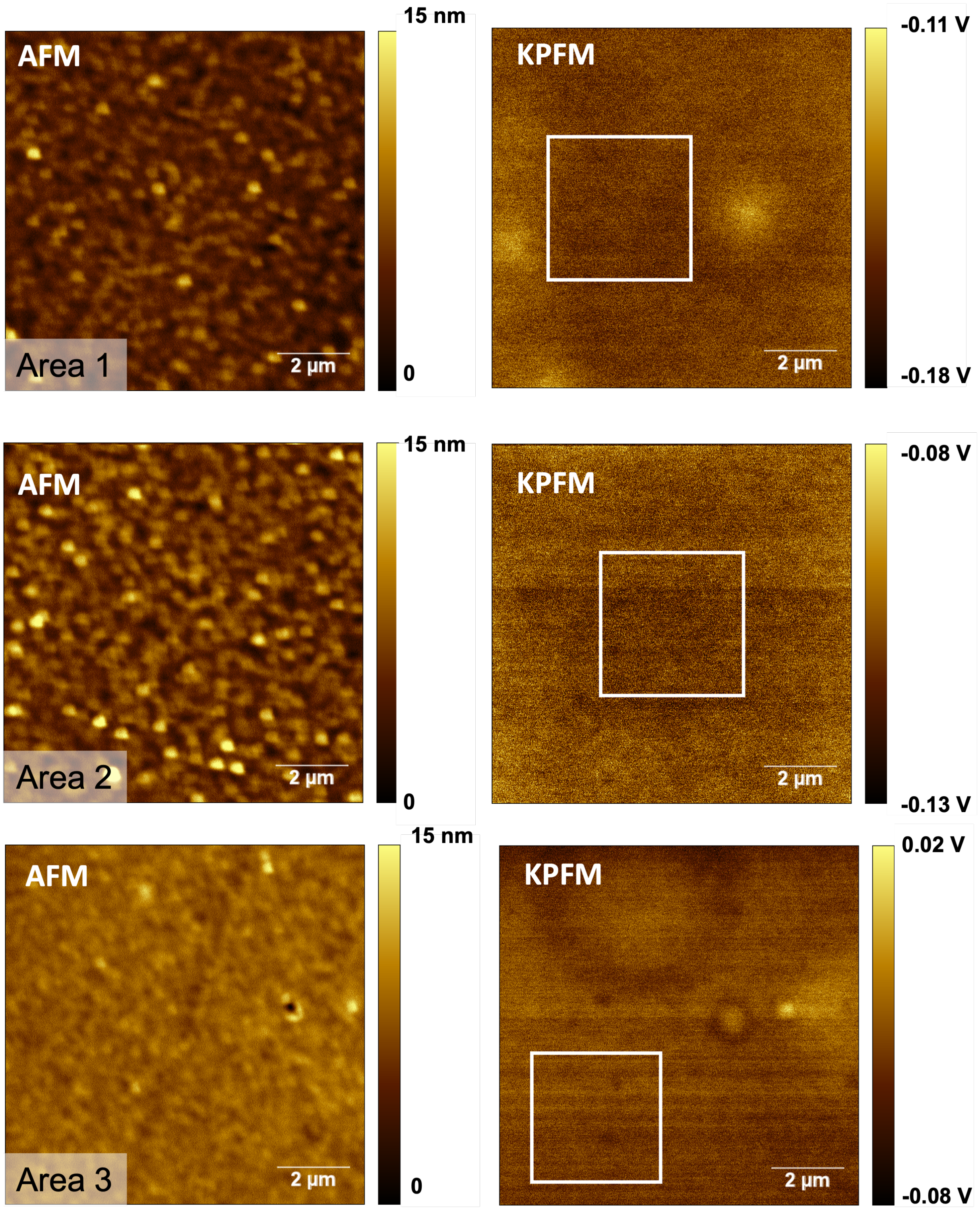

Figure S16: AFM (left) and KPFM (right) scan images of three selected areas on a 15s sulfurized Cu_2S film.

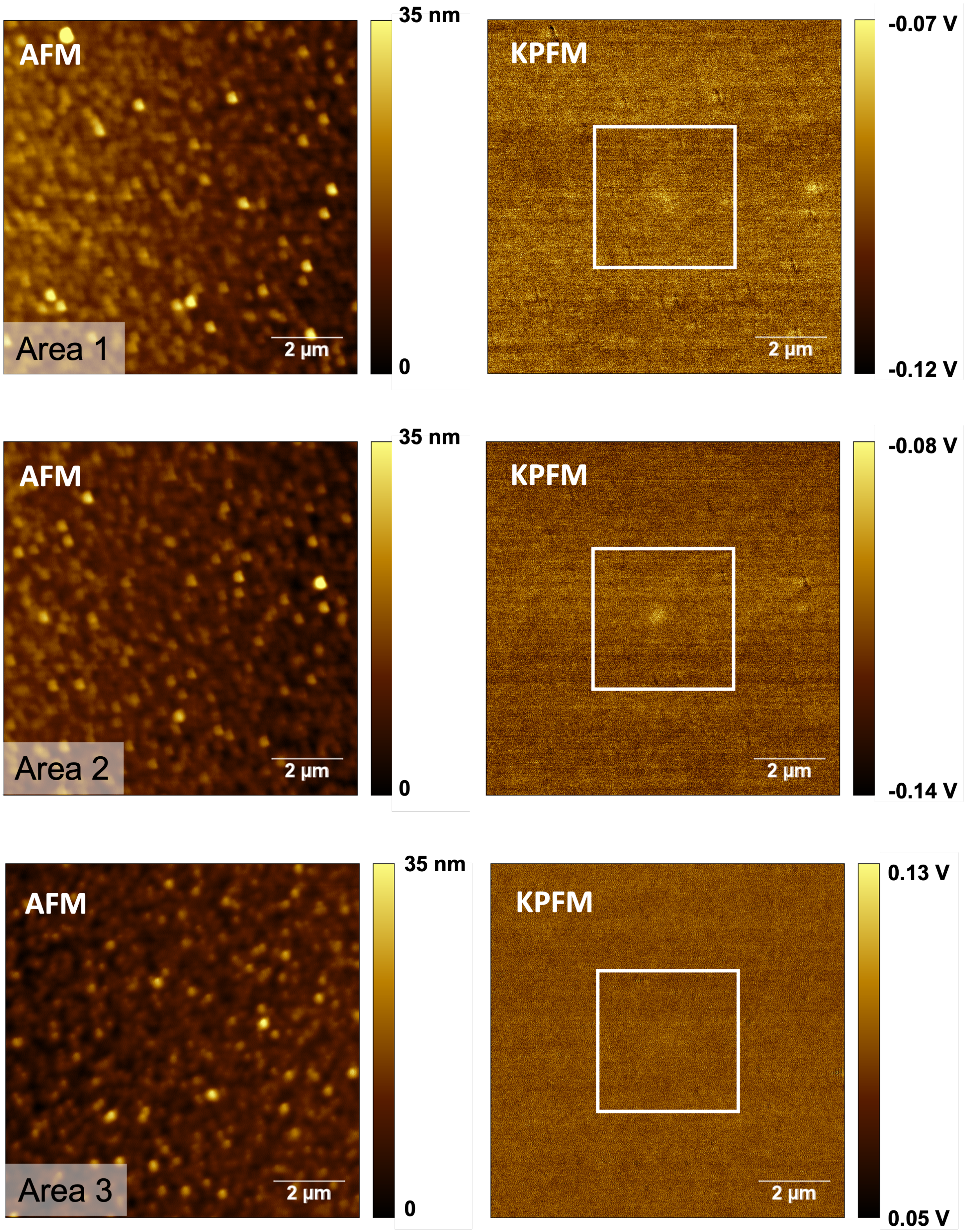

Figure S17: AFM (left) and KPFM (right) scan images of three selected areas on a 20s sulfurized Cu_2S film.

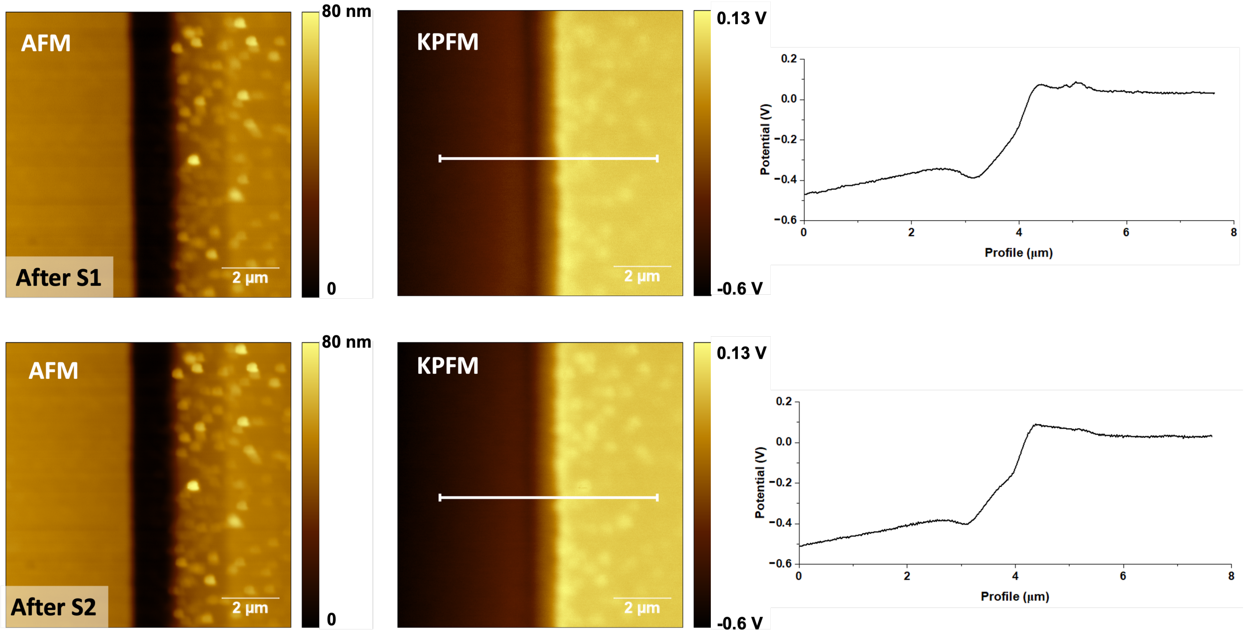

Figure S18: AFM (left) and KPFM (middle) scan images and potential profile (right) of Al and Au on silicon reference sample after 5s (S1) and 10s (S2) sulfurized Cu_2S film.

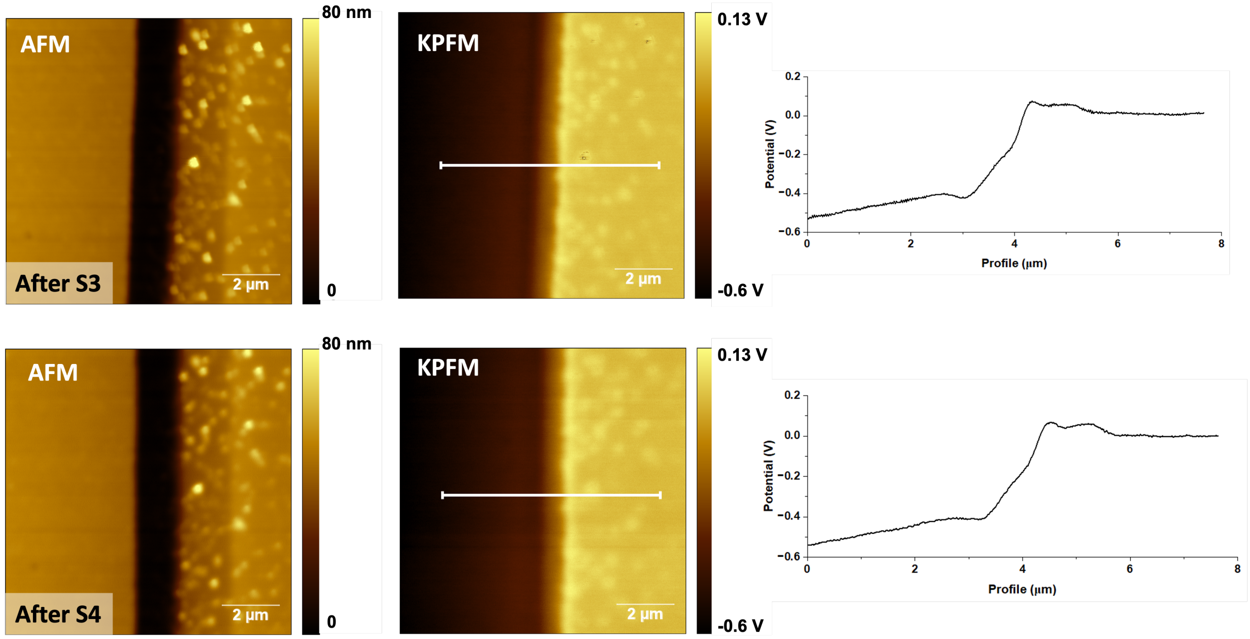

Figure S19: AFM (left) and KPFM (middle) scan images and potential profile (right) of Al and Au on silicon reference sample after 15s (S3) and 20s (S4) sulfurized Cu_2S film.

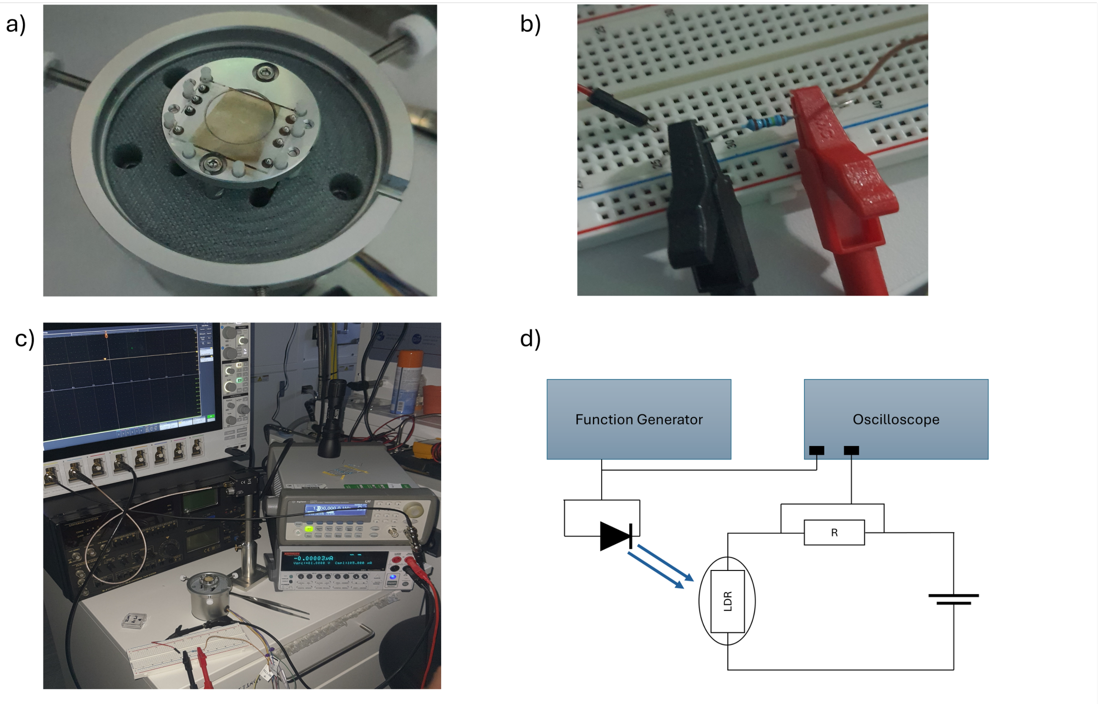

Figure S20: (a) Cu_2S device mounted on a homemade pin-contacted measurement holder. (b) Resistor connected in series with the sample holder, with the oscilloscope connected in parallel via crocodile clips. (c) Snapshot image of the experimental setup. (d) Circuit diagram used for electrical measurements of the Cu_2S device.

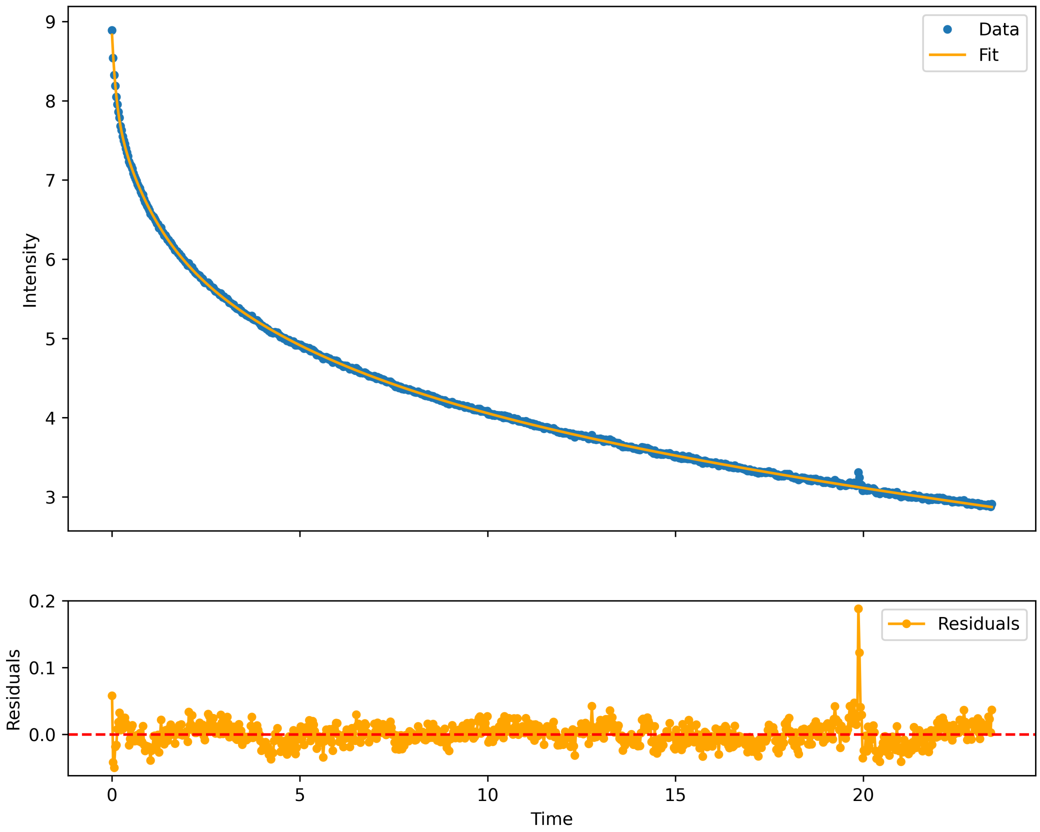

Figure S21: CTR as a function of exposure time for the Cu_2S device.

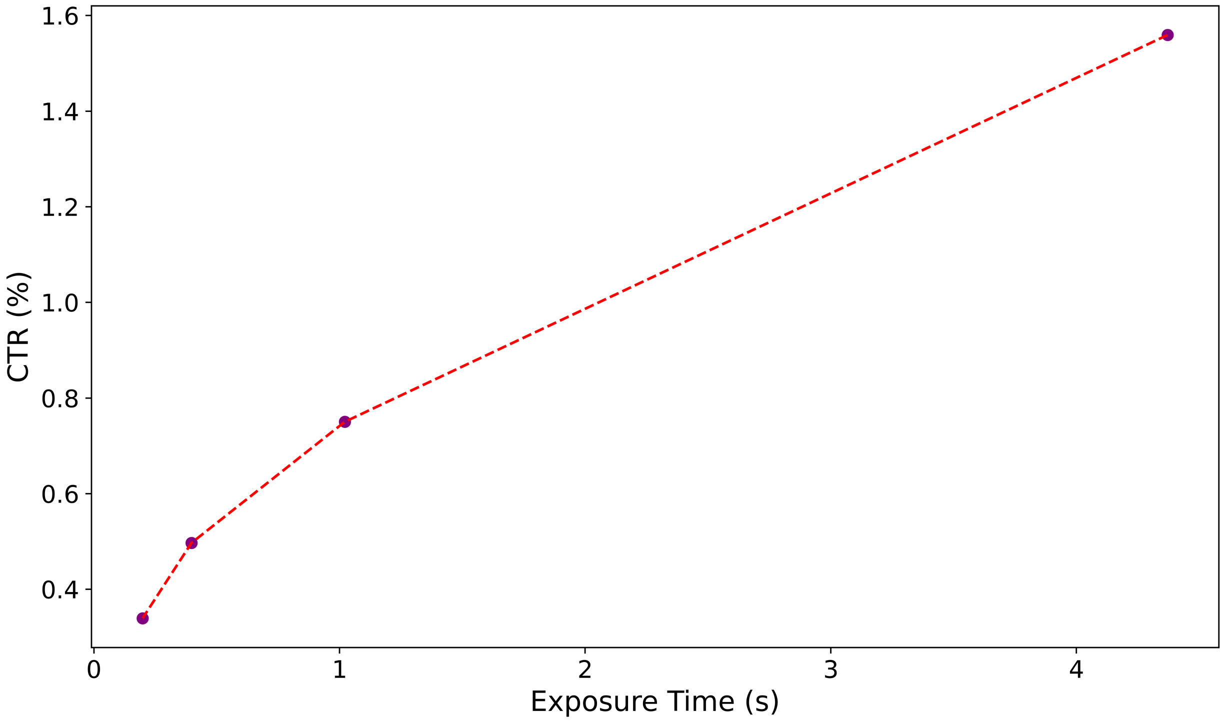

Figure S22: Representative quad-exponential decay fit applied to the transient decay curves of the Cu_2S device shown in Figure 5c of the main text.

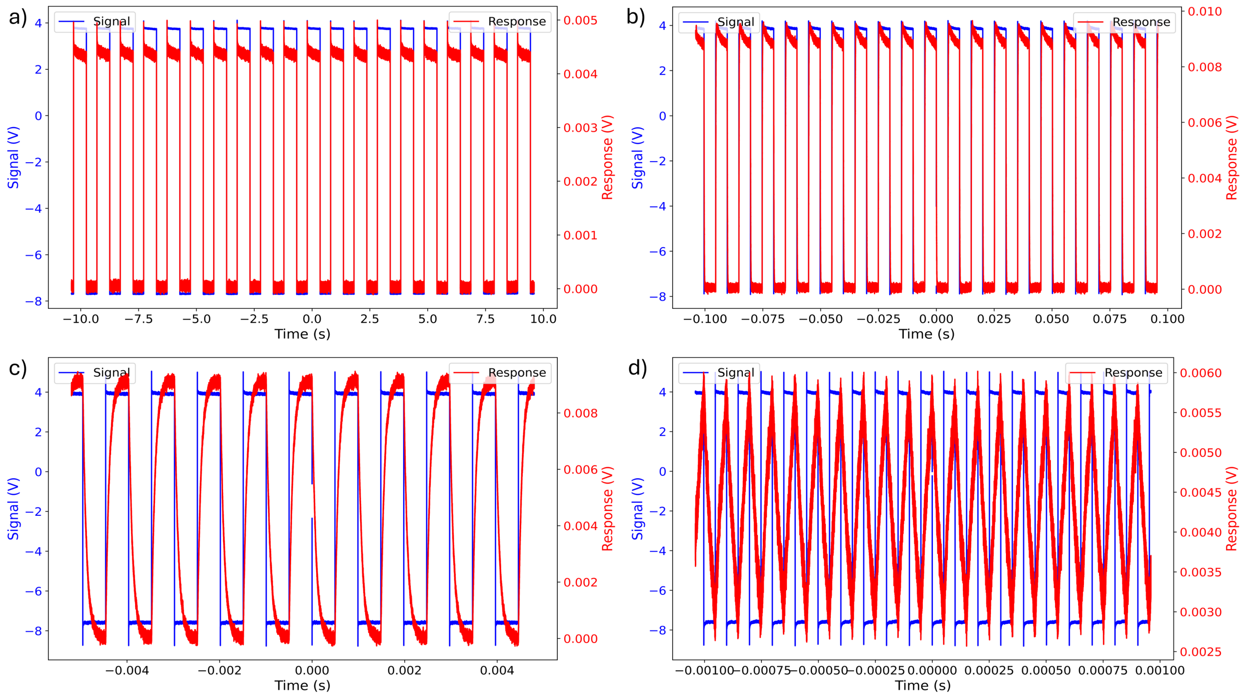

Figure S23: Representative electrical waveforms applied to drive the LED used for Cu_2S device measurements, together with the corresponding response of a reference Si photodiode used to validate the measurement setup. The LED was modulated at (a) 1 Hz, (b) 100 Hz, (c) 1 kHz, and (d) 10 kHz.

References

[1] Sansregret, J. L. Reaction of Copper Oxide with H 2 S. *Journal of The Electrochemical Society* **1980**, *127*, 2083, Publisher: IOP Publishing.

[2] Sharma, S. P. Reaction of Copper and Copper Oxide with H 2 S. *Journal of The Electrochemical Society* **1980**, *127*, 21, Publisher: IOP Publishing.

[3] Paul, A.; Schwind, B.; Weinberger, C.; Tiemann, M.; Wagner, T. Gas Responsive Nanoswitch: Copper Oxide Composite for Highly Selective H2S Detection. *Advanced Functional Materials* **2019**, *29*, 1904505, _eprint: https://onlinelibrary.wiley.com/doi/pdf/10.1002/adfm.201904505.

[4] Berendsen, H. J. C.; van der Spoel, D.; van Drunen, R. GROMACS: A message-passing parallel molecular dynamics implementation. *Computer Physics Communications* **1995**, *91*, 43–56.

[5] Van Der Spoel, D.; Lindahl, E.; Hess, B.; Groenhof, G.; Mark, A. E.; Berendsen, H. J. C. GROMACS: Fast, flexible, and free. *Journal of Computational Chemistry* **2005**, *26*, 1701–1718, _eprint: https://onlinelibrary.wiley.com/doi/pdf/10.1002/jcc.20291.

[6] Heinz, H.; Vaia, R. A.; Farmer, B. L.; Naik, R. R. Accurate Simulation of Surfaces and Interfaces of Face-Centered Cubic Metals Using 126 and 96 Lennard-Jones Potentials. *The Journal of Physical Chemistry C* **2008**, *112*, 17281–17290, Publisher: American Chemical Society.

[7] Best, R. B.; Zhu, X.; Shim, J.; Lopes, P. E. M.; Mittal, J.; Feig, M.; MacKerell, A. D. J. Optimization of the Additive CHARMM All-Atom Protein Force Field Targeting Improved Sampling of the Backbone , and Side-Chain 1 and 2 Dihedral Angles. *Journal of Chemical Theory and Computation* **2012**, *8*, 3257–3273, Publisher: American Chemical Society.

[8] Malde, A. K.; Zuo, L.; Breeze, M.; Stroet, M.; Poger, D.; Nair, P. C.; Oostenbrink, C.; Mark, A. E. An Automated Force Field Topology Builder (ATB) and Repository: Version 1.0. *Journal of Chemical Theory and Computation* **2011**, *7*, 4026–4037, Publisher: American Chemical Society.

[9] Berendsen, H. J. C.; Grigera, J. R.; Straatsma, T. P. The missing term in effective pair potentials. *The Journal of Physical Chemistry* **1987**, *91*, 6269–6271, Publisher: American Chemical Society.

[10] Hanwell, M. D.; Curtis, D. E.; Lonie, D. C.; Vandermeersch, T.; Zurek, E.; Hutchison, G. R. Avogadro: an advanced semantic chemical editor, visualization, and analysis platform. *Journal of Cheminformatics* **2012**, *4*, 17.

[11] Verlet, L. Computer "Experiments" on Classical Fluids. I. Thermodynamical Properties of Lennard-Jones Molecules. *Physical Review* **1967**, *159*, 98–103, Publisher: American Physical Society.

[12] Whitten, J. E. Ultraviolet photoelectron spectroscopy: Practical aspects and best practices. *Applied Surface Science Advances* **2023**, *13*, 100384.
